# A Bayesian susceptible-infectious-hospitalized-ventilated-recovered model to predict demand for COVID-19 inpatient care in a large healthcare system

**DOI:** 10.1371/journal.pone.0260595

**Published:** 2022-12-15

**Authors:** Stella Coker Watson Self, Rongjie Huang, Shrujan Amin, Joseph Ewing, Caroline Rudisill, Alexander C. McLain

**Affiliations:** 1 Department of Epidemiology and Biostatistics, Arnold School of Public Health, University of South Carolina, Columbia, South Carolina, United States of America; 2 Care Coordination Institute, Prisma Health, Greenville, South Carolina, United States of America; International Prevention Research Institute, FRANCE

## Abstract

The COVID-19 pandemic has strained healthcare systems in many parts of the United States. During the early months of the pandemic, there was substantial uncertainty about whether the large number of COVID-19 patients requiring hospitalization would exceed healthcare system capacity. This uncertainty created an urgent need to accurately predict the number of COVID-19 patients that would require inpatient and ventilator care at the local level. As the pandemic progressed, many healthcare systems relied on such predictions to prepare for COVID-19 surges and to make decisions regarding staffing, the discontinuation of elective procedures, and the amount of personal protective equipment (PPE) to purchase. In this work, we develop a Bayesian Susceptible-Infectious-Hospitalized-Ventilated-Recovered (SIHVR) model to predict the burden of COVID-19 at the healthcare system level. The Bayesian SIHVR model provides daily estimates of the number of new COVID-19 patients admitted to inpatient care, the total number of non-ventilated COVID-19 inpatients, and the total number of ventilated COVID-19 patients at the healthcare system level. The model also incorporates county-level data on the number of reported COVID-19 cases, and county-level social distancing metrics, making it locally customizable. The uncertainty in model predictions is quantified with 95% credible intervals. The Bayesian SIHVR model is validated with an extensive simulation study, and then applied to data from two regional healthcare systems in South Carolina. This model can be adapted for other healthcare systems to estimate local resource needs.

## Introduction

The World Health Organization declared the COVID-19 outbreak a global pandemic on March 11th, 2020. COVID-19 is a respiratory disease caused by the SARS-CoV-2 virus, which spreads primarily from person to person through respiratory droplets [1]. The pandemic spread rapidly across the globe, with over 114,415,000 infections and 2,539,000 deaths reported worldwide by March 1st, 2021 [2]. The United States (US) accounted for approximately 25.0% and 20.2% of those cases and deaths, respectively [2], and over 1,800,000 individuals in the US had been hospitalized for COVID-19 by March 1st, 2021. In late winter and early spring of 2020, the rapid spread of COVID-19 threatened to overwhelm healthcare systems with more patients than the systems could accommodate. In the US, these concerns caused state governments to close nonessential businesses, issue stay at home orders, and mandate other forms of social distancing in an attempt to mitigate the spread of the virus. While such efforts initially prevented large outbreaks in many states, the pandemic nevertheless strained healthcare systems in some parts of the country, particularly in New York City. The gradual easing of these restrictions during the late spring and summer of 2020 caused a resurgence of COVID-19, straining inpatient and intensive care unit (ICU) capacity in several states, most notably in Texas and Arizona. A second surge took place in the late fall of 2020 and winter of 2020–2021, with many parts of the US experiencing daily death tolls three to four times higher than what they experienced during the summer surge [2]. A third surge driven by the Delta variant took place in the late summer and early fall of 2021 with significant morbidity and mortality.

It remains critically important to accurately predict local demand for COVID-19-related care, as well as to develop models that can be used to estimate the demand for inpatient care during future infectious disease outbreaks. Such predictions provide advance notice if the demand for inpatient or ventilator care is likely to exceed the available capacity. This advanced warning gives healthcare systems time to expand capacity by increasing staffing, purchasing more ventilators, converting regular beds to ICU beds, reducing elective procedures to leave more resources for COVID-19 patients, and, in extreme cases, constructing temporary field hospitals. Furthermore, appropriate uncertainty intervals are necessary to ensure that healthcare systems are prepared for worst case scenarios. Even when demand for COVID-19 inpatient care is unlikely to exceed the available capacity, such demand predictions are still valuable for making staffing decisions, determining the ideal capacity for designated ‘COVID-19 wards’, and estimating the amount of personal protective equipment needed. Vaccines for COVID-19 became available in December, 2020 and are likely to greatly reduce the burden of COVID-19 on the healthcare system. However, as the vaccines are not 100% effective [3–5], and a large portion of the US population is reluctant to be vaccinated [6], demand for inpatient COVID-19 care will continue. Subsequently, the need to predict the number of individuals requiring such care will persist for the foreseeable future.

A variety of models exist for predicting the burden of COVID-19. The Institute for Health Metrics and Evaluation (IHME) model was one of the most widely used models during the early months of the pandemic, but concerns over the accuracy of this initial model led to the development of an updated version later in 2020 [7, 8]. While the IHME model provides state level estimates for resource use, it does not provide estimates on the health system level, limiting its usefulness for local health system planning [7]. The University of Massachusetts Amherst model uses of a number of other models to create an ensemble model, which is robust to small fluctuations in observed data, but is again not customizable to the healthcare system level [9]. Numerous other models exist for predicting COVID-19 cases, hospitalizations, ventilations, and mortality. The Centers for Disease Control and Prevention (CDC) provides a helpful summary of existing prediction models on their webpage [10].

Among the many models for predicting the burden of COVID-19 are a number of compartmental models such as susceptible-infectious-recovered (SIR) and susceptible-exposed-infectious-recovered (SEIR) models. Such compartmental models consist of a system of differential equations which describes how the population moves among various disease compartments (susceptible, infectious, recovered, etc.). Some of these models are deterministic and estimate key parameters from data prior to fitting the model. Others attempt to find optimal parameters values by fitting model projections to observed data and quantifying the resulting uncertainty. The COVID-19 Hospital Impact Model for Epidemics (CHIME) is a deterministic SIR model which is customizable to the healthcare system level [11]. However, the CHIME model quantifies uncertainty in predictions via a sensitivity analysis which requires many assumptions about the relative likelihood of various parameter values rather than estimating the uncertainty directly from the observed data. Pei and Shaman (2020) use a deterministic metropolitan SEIR model which accounts for the movement of people between counties to forecast county-level COVID-19 incidence for the US [12]. The Differential Equations Leads to Predictions of Hospitalizations and Infections (DELPHI) model from the COVIDAnalytics group at the Massachusetts Institute of Technology is another deterministic SEIR model, with additional model states to account for undocumented infections, hospitalizations, and individuals in quarantine [13].

A major drawback of deterministic SIR and SEIR approaches is that they fail to appropriately quantify the uncertainty in their estimates. Quantifying the uncertainty in model projections of the utmost importance, as the trajectory of the COVID-19 pandemic is highly dynamic and sensitive to changes in social distancing, masking, and other societal dynamics. To make the best decisions and most appropriate contingency plans, healthcare systems need to quantify the relative likelihood of a range of possible scenarios, rather than basing decisions on a single point estimate of the number of individuals requiring care. Some models quantify uncertainty via a stochastic compartmental model. For example, the Johns Hopkins University Infectious Disease Dynamics COVID-19 Working Group’s COVID Scenario Pipeline includes a SEIR model in which the transitions from one compartment to another are simulated from a binomial distribution [14]. Another way to improve the uncertainty quantification of compartmental models is to embed a SIR or SEIR system of differential equations inside a Bayesian statistical model. This approach allows one to combine the predictive capabilities of the SIR/SEIR model with the tractable inference framework of the Bayesian paradigm. De Brouwer et al. (2020) use a Bayesian SIER model to estimate the effectiveness of various COVID-19 containment measures implemented in different counties [15]. Mbuvha and Marwala (2020) use Bayesian SIR and SEIR models to estimate the total number of COVID-19 cases in South Africa, with particular attention paid to the Markov chain Monte Carlo techniques needed for fitting such models [16]. Alternately, de Oliveira et al. (2020) utilize a Bayesian SIR model to estimate the rate of COVID-19 underreporting in Brazil [17]. Hidaka and Torii (2020) use a Bayesian SIR model to predict the number of COVID-19 cases in the US and several other countries [18]. However, none of these Bayesian compartmental models allow for local hospital resource use estimation.

In this paper, we present a Bayesian susceptible-infectious-hospitalized-ventilated-recovered (SIHVR) model; this model is similar to the standard SIR model, but includes additional states for hospitalization and ventilation. Our model is designed to make predictions at the healthcare system level. Our model predicts the daily number of reported COVID-19 cases, the daily number of COVID-19 inpatient admissions, the daily census of hospitalized, non-ventilated of COVID-19 patients, and the daily census of ventilated COVID-19 patients. These quantities are assumed to follow negative binomial distributions whose means are governed by the solution to the SIHVR system of differential equations. The Bayesian framework allows us to estimate model parameters from the observed data and quantify the uncertainty in our parameter estimates. To our knowledge, our model is the only existing Bayesian SIHVR model for predicting the burden of COVID-19 at the healthcare system level that includes time varying transmission and hospitalization rates, and simultaneously utilizes hospital admissions data, inpatient census data, and ventilated census data, as well as the number of reported COVID-19 cases and social distancing metrics at the county level. We describe our model in the second section, demonstrate its performance via an extensive simulation study in the third section, and evaluate its real-world predictive performance using data from the largest healthcare system in South Carolina in the fourth section. The fifth section provides concluding remarks.

## Methodology

### The data

Our Bayesian SIHVR model integrates data from Prisma Health, the South Carolina Department of Health and Environmental Control (SCDHEC), the New York Times COVID-19 GitHub and Unacast’s social distancing metrics (derived from mobile phone GPS location data). Prisma Health is the largest healthcare provider in South Carolina and includes two major regional systems, Prisma Upstate and Prisma Midlands, which serve patient populations in geographically distinct parts of the state. The daily number of SARS-CoV-2 positive inpatient admissions to each regional system was obtained from March 6th, 2020 (the date of the first reported case of COVID-19 in South Carolina) to March 6th, 2021. The total number of SARS-CoV-2 positive individuals in inpatient care and not on a ventilator (non-ventilated census) and total number of SARS-CoV-2 positive individuals on a ventilator (ventilated census) was obtained for each system over the same time period.

The number of confirmed new SARS-CoV-2 infections reported each day was obtained from SCDHEC during the early months of the pandemic. These cases are aggregated to the county level, and publicly available [19]. A confirmed case is defined as an individual who had a positive polymerase chain reaction or antigen test for SARS-CoV-2 conducted via nose or throat swab, regardless of whether the individual had symptoms of infection or not. The reporting date reflects the date the case was publicly announced by SCDHEC. Data for the winter surge (November 2021 to March 2021) was obtained from the New York Times Github repository, as SCDHEC stopped providing daily COVID-19 updates in 2021 [20].

Social distancing data was obtained from the Unacast Social Distancing Scoreboard (USDS). Specifically, we used the ‘Change in Non-Essential Visits’ metric, which estimates the daily percent change in visits to non-essential businesses over pre-pandemic levels (defined as the 4 week period prior to March 8th, 2020) by tracking GPS locations from mobile phones [21].

Not all SARS-CoV-2 infections are documented by a positive viral test for SARS-CoV-2. Evidence from SARS-CoV-2 antibody tests suggests there were 6–24 unreported infections for each reported infection during the early months of the pandemic [22]. Reliable data on antibody seroprevalence over time are currently not available for South Carolina. As a result, we use the daily number of confirmed COVID-19 cases reported each day. The bias in the number of confirmed versus actual cases has led to the development of variants of the SIR model (e.g. Hao et al., 2020 [23]), which aim to estimate the number of actual cases over time using assumptions about dynamics of COVID-19 transmission. Here, our goal is to predict the number of SARS-CoV-2 positive patients requiring inpatient and ventilator care, not the incubation period, the reported number of cases, or the actual number of SARS-CoV-2 positive individuals, which are further upstream than our quantities of interest. As a result, the bias in the number of confirmed infections is not an issue as long as we can relate the number of confirmed cases to the number of cases requiring inpatient and ventilator care.

Another issue is that the available data consists of the number of confirmed or suspected COVID-19 cases requiring inpatient or ventilator care, which may differ from the true number of such cases. Fortunately, most COVID-19 cases requiring hospitalization are documented with a positive SARS-CoV-2 viral test, as testing anyone presenting with COVID-19-like symptoms for active SARS-CoV-2 infection has become standard practice. As a result, almost all hospitalized cases of COVID-19 are documented with a positive test result, and underdetection of hospitalized cases is not of major concern. We examine the effects of underdetection in case incidence via simulation in study in the next section, and find that while underdetection does impede our model’s ability to accurately estimate the number of cases in a given county, its effect on the model’s ability to estimate the number of patients hospitalized and ventilated (of primary interest here) is minimal.

### The data likelihood

Let *U*_*ct*_ denote the number of COVID-19 cases reported from area *c* on day *t*, for *c* = 1, 2, …, *C*, and *t* = 1, 2, …, *T*, where *C* is the number of areas (e.g. counties, census tracts, etc.) served by the healthcare system in question, and *T* is the number of days of data we wish to include in the model. Furthermore, let *W*_*t*_ denote the number of COVID-19 patients admitted to inpatient care on day *t*, *Y*_*t*_ denote the total number of hospitalized, non-ventilated patients on day *t*, and *Z*_*t*_ denote the total number of ventilated patients on day *t*. We assume:

$$\begin{array}{c} U_{ct}∼\text{Negative}\;\text{Binomial}(\mu_{i_{c}t},\sigma_{i}),\;\text{for}\;c=1,2,\ldots ,C,t=1,2,\ldots ,T \end{array}$$ (1)

$$\begin{array}{c} W_{t}∼\text{Negative}\;\text{Binomial}(\mu_{at},\sigma_{a})\;\text{for}\;t=1,2,\ldots ,T \end{array}$$ (2)

$$\begin{array}{c} Y_{t}∼\text{Negative}\;\text{Binomial}(\mu_{ht},\sigma_{h})\;\text{for}\;t=1,2,\ldots ,T \end{array}$$ (3)

$$\begin{array}{c} Z_{t}∼\text{Negative}\;\text{Binomial}(\mu_{vt},\sigma_{v})\;\text{for}\;t=1,2,\ldots ,T \end{array}$$ (4)

where *X* ∼ Negative Binomial(*μ*, *σ*) indicates that the random variable *X* follows a negative binomial distribution with mean *μ* and size *σ*, where $\mu_{i_{c}t}$, *μ*_*at*_, *μ*_*ht*_, and *μ*_*vt*_ are determined by the SIHVR system of differential equations specified in detail below and *σ*_*i*_, *σ*_*a*_, *σ*_*h*_ and *σ*_*v*_ are non-negative. Furthermore, we assume $U_{ct}|\mu_{i_{c}t}\sigma_{i},W_{t}|\mu_{at},\sigma_{a},Y_{t}|\mu_{ht},\sigma_{h}\;\text{and}\;Z_{t}|\mu_{vt},\sigma_{v}$ are independent for all *c* = 1, …, *C* and *t* = 1, …, *T*. We note that the *actual* number of newly infected individuals each day, conditional on its mean, is likely not independent of the number of hospitalized individuals on subsequent days (as a higher than predicted number of new infections on a given day is likely to trigger a higher than expected number of hospitalizations in subsequent days). However, recall that *U*_*ct*_ is the *reported* number of new infections. Random fluctuations in the number of reported cases can be due to a number of factors besides fluctuations in the actual number of newly infected individuals, such as reporting delays, slower lab processing times over the weekends, changes in testing availability, etc. Thus, much of the day to day fluctuation in the number of reported cases does not directly correspond to a similar fluctuation in the actual number of newly infected individuals, and assuming the number of hospitalized individuals and the number of reported cases are temporally independent after conditioning on their means is not unreasonable. In fact, we found that attempts to account for the potential temporal dependence between the $U_{ct}|\mu_{i_{c}t}\sigma_{i}\text{s}$ and the *Y*_*t*_|*μ*_*ht*_*σ*_*h*_s did not improve the predictive performance of the model.

### The SIHVR model

We assume the population of each area is composed of susceptible, infectious (but not hospitalized), hospitalized (but not ventilated), ventilated, and recovered individuals. Let *N*_*c*_ denote the population of area *c*, and let *S*_*c*_(*t*), *I*_*c*_(*t*), *H*_*c*_(*t*), *V*_*c*_(*t*), and *R*_*c*_(*t*) denote the number of susceptible individuals, infectious non-hospitalized individuals, hospitalized non-ventilated individuals, ventilated individuals, and recovered individuals from area *c* at time *t*, respectively. As we are not interested in predicting the number of COVID-19 deaths, we treat dead and recovered patients as single category (recovered). While it is possible for individuals who have recovered from COVID-19 to become reinfected, preliminary evidence suggests most recovered individuals are immune for at least 6–8 months [24, 25]. We are primarily interested in using 1–5 months of data to produce short-term predictions (2–4 weeks in the future). As individuals are unlikely to be reinfected during a 5 month time period, reinfections are not a major concern for our purposes. Finally, our model was developed prior to the availability of COVID-19 vaccines, and thus we do not directly account for the effects of vaccines in our model. However, the effect of vaccinations is indirectly accounted for by allowing the disease transmission rate to vary over time. As data on the number of vaccinated individuals becomes available, our method can easily be extended directly account for the effect of vaccines by allowing individuals to move from the susceptible state to a ‘vaccinated’ state with reduced susceptibility to infection.

**Fig 1:**
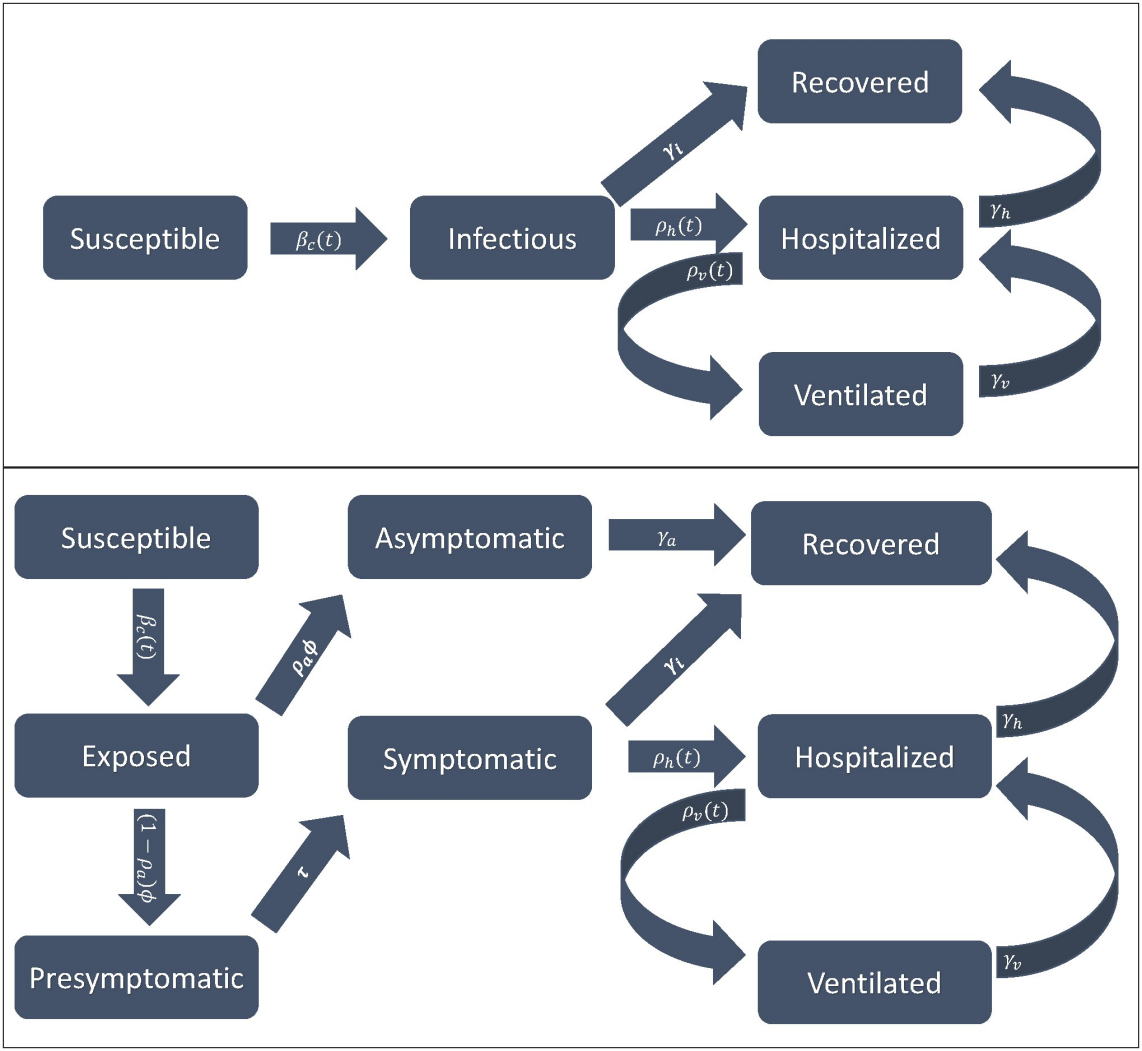
An illustration of the SIHVR model (top) and SEAPSHVR model (bottom). Rectangles represent compartments, and arrows indicate the flow of individuals between compartments.

We assume that the number of individuals in each state at time *t* is governed by the solution to the following system of differential equations:

$$\begin{array}{c} \frac{dS_{c}}{dt}=-\beta_{c}\left(t\right)I_{c}\left(t\right)S_{c}\left(t\right)/N_{c} \end{array}$$ (5)

$$\begin{array}{c} \frac{dI_{c}}{dt}=\beta_{c}\left(t\right)I_{c}\left(t\right)S_{c}\left(t\right)/N_{c}-\gamma_{i}I_{c}\left(t\right)-\rho_{h}\left(t\right)I_{c}\left(t\right) \end{array}$$ (6)

$$\begin{array}{c} \frac{dH_{c}}{dt}=\rho_{h}\left(t\right)I_{c}\left(t\right)-\gamma_{h}H_{c}\left(t\right)-\rho_{v}\left(t\right)H_{c}\left(t\right)+\gamma_{v}V_{c}\left(t\right) \end{array}$$ (7)

$$\begin{array}{c} \frac{dV_{c}}{dt}=\rho_{v}\left(t\right)H_{c}\left(t\right)-\gamma_{v}V_{c}\left(t\right) \end{array}$$ (8)

$$\begin{array}{c} \frac{dR_{c}}{dt}=\gamma_{i}I_{c}\left(t\right)+\gamma_{h}H_{c}\left(t\right) \end{array}$$ (9)

for *c* = 1, …, *C* and *t* ∈ [0, *T*]. Here *γ*_*i*_ ∈ (0, 1) is the recovery rate for non-hospitalized individuals, *γ*_*h*_ ∈ (0, 1) is the discharge rate of non-ventilated individuals, and *γ*_*v*_ ∈ (0, 1) is the rate at which ventilated individuals are removed from the ventilator. The transmission rate for area *c* (*β*_*c*_(*t*)), the proportion of infected people entering the hospital each day (*ρ*_*h*_(*t*)), and the proportion of hospitalized patients beginning ventilation each day (*ρ*_*v*_(*t*)) are time-varying. We assume they have the following form:

$$\begin{array}{c} \beta_{c}\left(t\right)=\text{exp}\left\{\eta_{c}\left(t\right)\right\}\;\eta_{c}\left(t\right)=x_{i_{c}t}^{\prime }b_{i}+z_{ct}^{\prime }d+\alpha_{c}\;\text{for}\;c=1,\ldots ,C \end{array}$$

$$\begin{array}{c} \rho_{h}\left(t\right)=g\left\{\eta_{h}\left(t\right)\right\},\;\eta_{h}\left(t\right)=x_{ht}^{\prime }b_{h} \end{array}$$

$$\begin{array}{c} \rho_{v}\left(t\right)=g\left\{\eta_{v}\left(t\right)\right\},\;\eta_{v}\left(t\right)=x_{vt}^{\prime }b_{v} \end{array}$$

where $x_{i_{c}t}$, ***x***_***ht***_, and ***x***_***vt***_ are B-spline basis functions in time evaluated at *t*, ***b***_*i*_, ***b***_*h*_ and ***b***_*v*_ are the associated vectors of coefficients, ***z***_*ct*_ is a vector of social distancing metrics from area *c* associated with day *t*, ***d*** are the associated coefficients, *α*_*c*_ is a random effect for area *c* and *g*(⋅) is the logistic function. For more on B-splines, see De Boor (1978) [26]. Fig 1 provides an illustration of the various model compartments and the flow of individuals between them. For more on SIR and other compartmental models, see Brauer et al. (2008) [27] or Tolles and Luong (2020) [28].

The above model results in $\mu_{i_{c}t}=\beta_{c}(t)I_{c}(t)S_{c}(t)/N_{c}$, that is, the mean number of new confirmed SARS-CoV-2 infections in area *c* on day *t* is equal to the rate of new infections in area *c* on day *t*. We also assume $\mu_{at}=\rho_{h}\left(t\right)\sum_{c=1}^{C}I_{c}\left(t\right)$, that is, the mean number of SARS-CoV-2 positive individuals admitted to inpatient care on day *t* is given by rate of flow into the hospitalization state on day *t*, summed over all areas. Finally, we assume $\mu_{ht}=\sum_{c=1}^{C}H_{c}\left(t\right)$, that is, the mean number of non-ventilated individuals in inpatient care on day *t* is equal to the total number of individuals in the non-ventilated state on day *t*, and $\mu_{vt}=\sum_{c=1}^{C}V_{c}\left(t\right)$, the mean number of ventilated individuals on day *t* is equal to the total number of individuals in the ventilated state at time *t*.

The proposed SIHVR model makes several simplifying assumptions about the transmission dynamics of SARS-CoV-2. In the model, individuals pass directly from the susceptible to the infectious state. However in reality, individuals do not immediately become contagious after acquiring SARS-CoV-2 infection [29]. Additionally, the SIHVR model does not differentiate between asymptomatic, presymptomatic, and symptomatic infections. However, asymptomatic and presymptomatic infections are known to influence the transmission of SARS-CoV-2 in practice [30, 31]. We assess the impact of these simplifying assumptions as part of the simulation study presented in the next section. Specifically, we generate data from a Susceptible-Exposed-Asymptomatic-Presymptomatic-Symptomatic-Hospitalized-Ventilated-Recovered (SEAPSHVR) model which accounts for these factors. In the SEAPSHVR model, the exposed state compromises individuals who have acquired infection but are not yet contagious, the asymptomatic state comprises infectious individuals who are asymptomatic and will remain so for the duration of their infection, the presymptomatic state comprises infectious individuals who will go on to develop symptoms, but have not done so yet, and the symptomatic state comprises symptomatic infectious individuals. Only symptomatic infections feed into the hospitalized state. A schematic of the SEAPSHVR model is shown in Fig 1, and a full description of this model can be found in Web Appendix B in S1 File.

### Initial conditions

It is also necessary to specify the initial conditions for the SIHVR system of differential equations. We assume the number of initially infected individuals is unknown, and estimate it along with the other parameters. The number of initially non-ventilated, hospitalized patients and ventilated patients are assumed to be known constants, as these can be confidently obtained from available data. The number of initially recovered individuals is also assumed to be known, and can be estimated from the number of previously documented infections and the estimated ratio of documented to undocumented cases for a particular area. Finally, we assume individuals who are not initially infectious, hospitalized, ventilated, or recovered are initially susceptible.

### Prior distributions

To fully specify our Bayesian model, it is necessary to assign prior distributions to all unknown parameters. We assume the following weakly informative prior distributions:

$$\begin{array}{cc} b_{i} & ∼N(0,\sigma_{b_{i}}^{2}I)\\ b_{h} & ∼N(0,\sigma_{b_{h}}^{2}I)\\ b_{v} & ∼N(0,\sigma_{b_{v}}^{2}I)\\ d & ∼N(0,\sigma_{d}^{2})\\ \gamma_{i},\gamma_{h},\gamma_{v} & ∼U(0,1)\\ \sigma_{i} & ∼TN(\mu_{\sigma_{i}},\sigma_{\sigma_{i}}^{2},0,\infty )\\ \sigma_{a} & ∼TN(\mu_{\sigma_{a}},\sigma_{\sigma_{a}}^{2},0,\infty )\\ \sigma_{h} & ∼TN(\mu_{\sigma_{i}},\sigma_{\sigma_{h}}^{2},0,\infty )\\ \sigma_{v} & ∼TN(\mu_{\sigma_{i}},\sigma_{\sigma_{v}}^{2},0,\infty )\\ \alpha & ∼N(0,\sigma_{\alpha }^{2}I)\\ \sigma_{\alpha }c^{2} & ∼IG(\alpha_{\alpha },\beta_{\alpha })\\ \;\;\;\;\;\;\;\;\;I_{c}\left(0\right) & ∼TN(\mu_{I_{c}\left(0\right)},\sigma_{I_{c}\left(0\right)}^{2},0,N_{c}),\;\text{for}\;c=1,2,\ldots ,C. \end{array}$$

Here ***X*** ∼ *N*(***μ***, ***A***) denotes that the random vector *X* follows a multivariate normal distribution with mean vector ***μ*** and variance-covariance matirx ***A***, *X* ∼ *U*(*a*, *b*) denotes that the random variable *X* follows a uniform distribution on the interval (*a*, *b*), *X* ∼ *TN*(*μ*, *σ*^2^, *a*, *b*) denotes that the random variable *X* follows a truncated normal distribution supported on the interval (*a*, *b*) with mean *μ*, and variance *σ*^2^, *X* ∼ *IG*(*a*, *b*) denotes that the random variable *X* follows an inverse gamma distribution with shape parameter *a* and scale parameter *b*, ***α*** = (*α*_1_, *α*_2_, …, *α*_*C*_)′, $\sigma_{b_{i}}^{2}=\sigma_{b_{h}}^{2}=\sigma_{b_{v}}^{2}=\sigma_{d}^{2}=\sigma_{\sigma_{i}}^{2}=\sigma_{\sigma_{a}}^{2}=\sigma_{\sigma_{h}}^{2}=\sigma_{\sigma_{v}}^{2}=\sigma_{I_{c}\left(0\right)}^{2}=1000$, $\mu_{\sigma_{i}}=\mu_{\sigma_{a}}=\mu_{\sigma_{h}}=\mu_{\sigma_{v}}=1$, *μ*_*I*_*c*_(0)_ = 4, and *α*_*α*_ = *β*_*α*_ = 2. We have elected to use weakly informative prior distributions to avoid making unnecessary assumpations and to ensure our model is accessible to healthcare systems who may lack the statistical expertise to select appropriate informative priors for their patient population.

### Model fitting procedure

We developed a Markov chain Monte Carlo (MCMC) algorithm to generate a sample of the unknown parameters from the posterior distribution. As the full conditional distributions of the model parameters are not recognizable, the sampling routine consists of Metropolis Hasting steps. For the full sampling algorithm, see Web Appendix A in S1 File. After generating a posterior parameter sample of size *G*, the SIHVR system of differential equations was solved *G* times, once for each set of sampled parameters, thereby generating a posterior sample of $\mu_{i_{c}t}$, *μ*_*at*_, *μ*_*ht*_, and *μ*_*vt*_, for *c* = 1, 2, …, *C* and *t* = 1, 2, …, *T*. As the distribution of these parameters was right skewed, the posterior median was used as point estimate, rather than the posterior mean. This sample, along with the posterior sample of *σ*_*i*_, *σ*_*a*_, *σ*_*h*_, and *σ*_*v*_, was used to generate a sample from the posterior predictive distribution of *U*_*ct*_, *W*_*t*_, *Y*_*t*_ and *Z*_*t*_, for *c* = 1, 2, …, *C* and *t* = 1, 2, …*T*. Specifically, for each value of *c* and *t* and for each pair $\left(\mu_{i_{c},t}^{\left(g\right)},\sigma_{i}^{\left(g\right)}\right)$ in the posterior sample, *g* = 1, 2, …, *G*, $U_{ct}^{\left(g\right)}$ was generated from a negative binomial distribution with mean $\mu_{i_{c}t}^{\left(g\right)}$ and size $\sigma_{i}^{\left(g\right)}$. Posterior samples for the *W*_*t*_s, *Y*_*t*_s and *Z*_*t*_s were generated analogously using the appropriate distributions. These samples were then used to create posterior prediction intervals for each quantity in the usual way. The MCMC algorithm was run for 20,000 iterations, with the first 10,000 iterations discarded as the burn in period. Convergence was assessed via trace plots.

## Simulation study

In this section, we evaluate the performance of the Bayesian SIHVR model with a simulation study. We first consider the case of perfect model specification with no underreporting of cases, and then evaluate model performance in the presence of model misspecification and/or underreporting of cases. For each of these scenarios, we evaluate ‘lockdown phase’ performance using *T* = 57 days of data (the equivalent of using data from the first SCDHEC reported case on March 6th to May 1st) and ‘surge phase’ performance, using *T* = 118 days of data (the equivalent of using data from March 6th to July 1st). For each dataset, the data from days *t* = 1, …, *T* was used to fit the model, and data from days *t* = *T* + 1, …, *T* + 14 was used to assess out of sample prediction performance.

To generate data under the assumption of perfect model specification, we take *C* = 2 areas, and solve the system of differential equations given by (5)–(9). The transmission rate and hospitalization rate are chosen so that the observed number of cases and hospitalizations are similar to the observed data from Prisma Upstate during the time period under consideration (March 6th, 2020 to May 1st, 2020 for the lockdown stage and March 6th, 2020 to July 1st, 2020 for the surge stage). As a result, these parameter specifications vary somewhat across data generation scenarios (for the specific conditions used for each of the scenarios, see Web Appendix B in S1 File). To mimic the noisy behavior of the social distancing metric, the observed USDS metric from Greenville and Spartanburg counties in the Upstate region of South Carolina was used as the social distancing metric in the transmission rate for data generation. A piecewise cubic B-spline basis with 4 (3) equally spaced knots was used to estimate the transmission (hospitalization) rate. We took ***z***_*ct*_ = *z*_*ct*−14_, the social distancing metric observed in county *c* on day *t* − 14, *ρ*_*v*_(*t*) = 0.05, *γ*_*i*_ = 1/14, *γ*_*h*_ = 1/10, *γ*_*v*_ = 1/10, *N*_1_ = 498402 and *N*_2_ = 302195 (the populations of Greenville and Spartanburg counties). After solving the system of differential equations given in (5)–(9), *U*_*ct*_, *W*_*t*_, *Y*_*t*_, and *Z*_*t*_, for *c* = 1, …, *C*, and *t* = 1, …, *T* + 14 were independently generated from Poisson distributions with means given by $\mu_{i_{c}t}=\beta_{c}(t)I_{c}(t)S_{c}(t)/N_{c}$, $\mu_{at}=\rho_{h}\left(t\right)\sum_{c=1}^{C}I_{c}\left(t\right)$, $\mu_{ht}=\sum_{c=1}^{C}H_{c}\left(t\right)$ and $\mu_{vt}=\sum_{c=1}^{C}V_{c}\left(t\right)$, respectively. For *T* ∈ {57, 118}, 500 independent datasets were generated as described above. To assess effect of underdetection under the assumption of perfect model specification, we generated 500 more datasets for each value of *T*, this time generating *U*_*ct*_ from a Poisson distribution with mean $\mu_{i_{c}t}\beta_{c}(t)I_{c}(t)S_{c}(t)/(10N_{c})$, that is, we assumed only 10% of cases are detected on average. For purposes of simplicity, we refer to these two scenarios as data generating mechanisms (DGMs) 1 and 2, respectively.

As previously mentioned, the transmission dynamics assumed by the SHIVR model are simpler than the true transmission dynamics of SARS-CoV-2. To assess the impact of these simplifying assumptions on the performance of our method, we performed additional simulations in which data was generated from the more complicated SEAPSHVR model introduced in the previous section. Under all three of these scenarios, the SIHVR model was misspecified, and there was some degree of case underdetection. First, to mimic the type of underdetection which occurs when testing is severely limited, *U*_*ct*_ was generated from a Poisson distribution with a mean equal to 10% of the rate of flow into the symptomatic infectious state from the presymptomatic infectious state on day *t* in county *c*, that is $\mu_{i_{ct}}=\tau P_{c}\left(t\right)/10$ (DGM 3, see Fig 1 and Web Appendix B in S1 File). Second, to assess method performance when testing is widely available to symptomatic individuals, *U*_*ct*_ was generated from a Poisson distribution with a mean equal to the rate of flow into the symptomatic infectious state from the presymptomatic infectious state on day *t* in county *c*, that is, $\mu_{i_{ct}}=\tau P_{c}\left(t\right)$ (DGM 4, see Fig 1 and Web Appendix B in S1 File). Finally, to assess performance when some asymptomatic infections are detected in addition to symptomatic infections, *U*_*ct*_ was generated from a Poisson distribution with mean equal to the rate of flow into the symptomatic infectious state from the presymptomatic infectious state plus 25% of the rate of flow into the asymptomatic state from the exposed state on day *t* in county *c*, that is, $\mu_{i_{ct}}=\tau P_{c}\left(t\right)+\rho_{a}\phi E_{c}\left(t\right)/4$ (DGM 5, see Fig 1 and Web Appendix B in S1 File). The specifications for all other SEAPSHVR model parameters under DGMs 3–5 can be found in Web Appendix B in S1 File.

**Fig 2:**
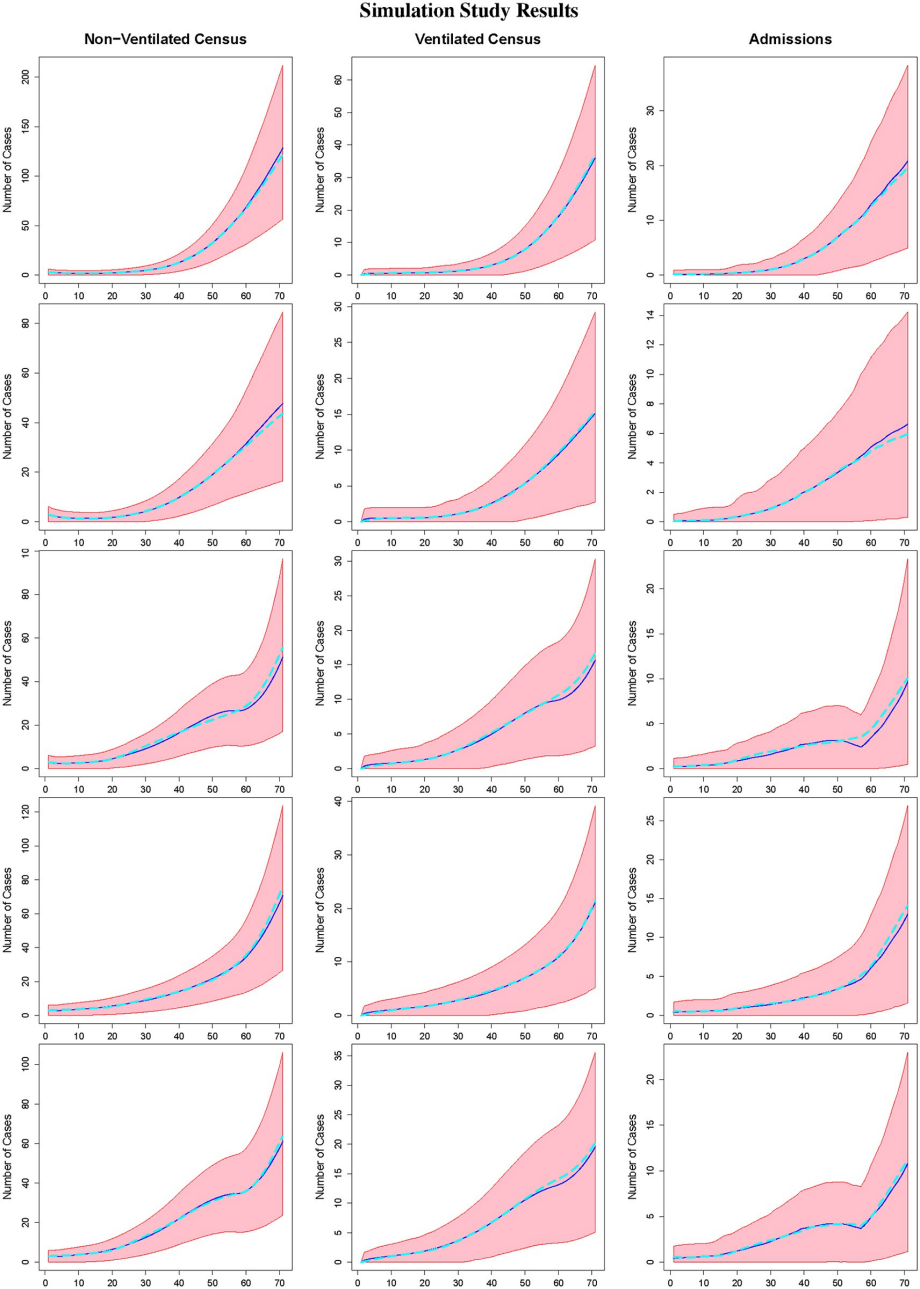
Simulation study results. The figure displays the posterior median (dark blue), true value used for data generation (light blue) and 95% prediction interval (red) for the non-ventilated census (column 1), ventilated census (column 2), and admissions (column 3). From top to bottom, the rows correspond with DGMs 1–5 with *T* = 57.

**Fig 3:**
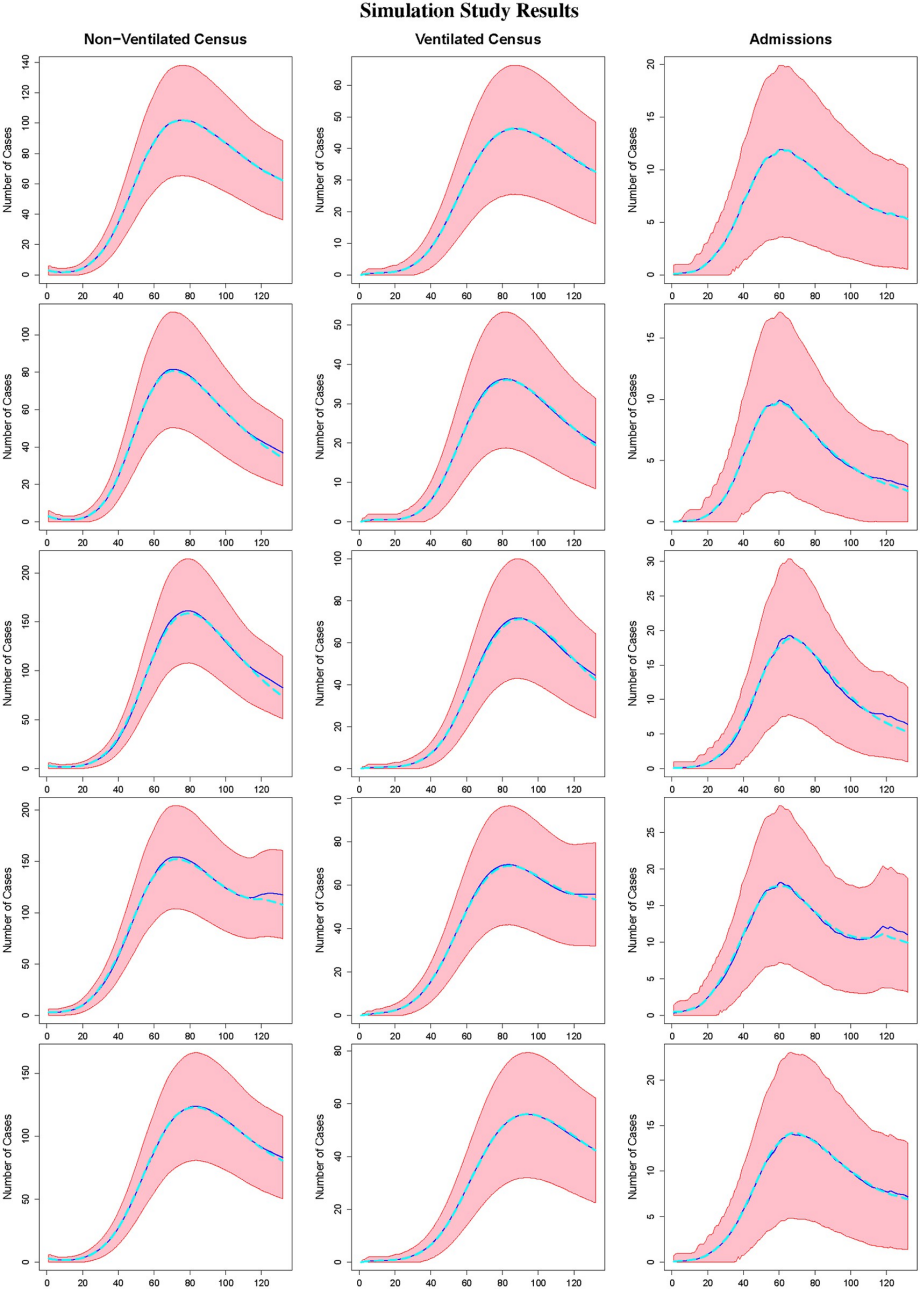
Simulation study results. The figure displays the posterior median (dark blue), true value used for data generation (light blue) and 95% prediction interval (red) for the non-ventilated census (column 1), ventilated census (column 2), and admissions (column 3). From top to bottom, the rows correspond with DGMs 1–5 with *T* = 118.

Tables 1 and 2 and Figs 2 and 3 summarize the results of our study. Specifically, Table 1 summarizes the results for the non-ventilated census, ventilated census, and hospital admissions. For each quantity, the table provides the estimated mean empirical bias, the mean absolute out of sample prediction error, the mean absolute out of sample prediction percent error, defined as the absolute bias divided by the true value multiplied by 100, and the empirical coverage probability of 95% prediction intervals based on out of sample data. The empirical bias is averaged over days *t* = 1, …, *T*, and the other quantities are averaged over days *t* = *T* + 1, …, *T* + 14. Web Table 1 provides similar information for the area-level reported case incidence. Figs 2 and 3 provide plots of the posterior median estimates, true values used for data generation, and 95% credible prediction intervals for the non-ventilated census, ventilated census, and new admissions over time, averaged over all 500 datasets for *T* = 57 and *T* = 118, respectively. Web Figs 1 and 2 display similar results for the estimated area-level reported case incidence and area-level transmission rates for *T* = 57 (lockdown) and *T* = 118 (surge), respectively. Note that as our primary goal is providing the healthcare system with a range of patient numbers requiring non-ventilated and ventilated inpatient care, the credible intervals are 95% credible intervals for *prediction*. Thus, we would expect roughly 95% of observations to fall within the interval, rather than expecting the interval to capture the true parameter value 95% of the time. Table 2 summarizes the results for key model parameters (*γ*_*h*_, *γ*_*v*_), providing the posterior mean estimate, and associated empirical bias, empirical MSE, empirical standard deviation, and empirical coverage probability (ECP) for 95% credible intervals. Web Table 2 provides the same information for *γ*_*i*_.

**Table 1:** Summary of simulation study results. The table provides the empirical bias (averaged over days 1, 2, …, *T* and the 500 datasets), empirical absolute prediction error (averaged over days *T* + 1, *T* + 2, …, *T* + 14 and 500 datasets), empirical absolute prediction percent error (averaged over days *T* + 1, *T* + 2, …, *T* + 14 and the 500 datasets), and empirical coverage probability for 95% prediction intervals (averaged averaged over days *T* + 1, *T* + 2, …, *T* + 14 and the 500 datasets).

Quantity	DGM*	Bias	Abs. Pred. Er.	% Abs. Pred. Er.	95% ECP**
	*Lockdown Phase (March 6 to May 1), T = 57*				
Non-Ventilated Census	1	0.0026	11.5243	12.38	0.9966
	2	0.0121	5.7292	15.42	0.9936
	3	0.0823	6.7267	16.56	0.9577
	4	0.0070	7.7039	14.47	0.9809
	5	0.0464	6.2673	12.99	0.9861
Ventilated Census	1	0.0124	3.0082	11.21	0.9940
	2	0.0156	1.6354	13.22	0.9861
	3	-0.0025	1.9316	14.68	0.9736
	4	-0.0148	1.9029	12.24	0.9887
	5	-0.0166	2.0906	12.69	0.9789
Admissions	1	0.0107	2.2893	14.63	0.9917
	2	-0.0013	1.1241	21.12	0.9836
	3	-0.1077	1.9453	28.92	0.9366
	4	-0.0436	1.9370	20.04	0.9673
	5	-0.0108	1.6480	21.46	0.9690
	*Surge Phase (March 6 to July 1), T = 118*				
Non-Ventilated Census	1	0.0455	3.1391	4.77	0.9974
	2	0.0908	2.9144	7.73	0.9943
	3	0.1987	7.1668	8.80	0.9989
	4	0.1734	7.9656	7.20	0.9984
	5	-0.0122	1.7153	3.82	0.9953
Ventilated Census	1	-0.0088	1.3656	3.94	0.9941
	2	0.0337	1.0460	4.89	0.9901
	3	0.0520	1.9364	4.15	0.9969
	4	0.0644	2.0731	3.81	0.9979
	5	-0.0409	0.6413	8.81	0.9826
Admissions	1	-0.0072	0.4573	8.11	0.9757
	2	-0.0089	0.4304	15.27	0.9867
	3	-0.0609	1.1501	19.33	0.9840
	4	0.0644	2.0731	3.81	0.9979
	5	-0.0409	0.6413	8.81	0.9826

*Data Generation Mechanism

**Empirical Coverage Probability

**Table 2:** Summary of simulation study results. The table provides the posterior mean estimate, empirical bias, MSE, standard deviation, and coverage probability for 95% credible intervals, averaged over all 500 datasets.

Parameter	DGM*	Estimate	Bias	MSE	SD	95% ECP**
	*Lockdown Phase (March 6 to May 1), T = 57*					
*γ*_*h*_	1	0.1017	0.0017	0.0005	0.0229	0.9580
	2	0.1009	0.0009	0.0005	0.0231	0.9500
	3	0.0920	-0.0080	0.0004	0.0173	0.9180
	4	0.0986	-0.0014	0.0003	0.0200	0.9620
	5	0.1007	0.0007	0.0002	0.0173	0.9700
*γ*_*v*_	1	0.4039	0.3039	0.1153	0.2486	0.7920
	2	0.4103	0.3103	0.1163	0.2556	0.8160
	3	0.4028	0.3028	0.1164	0.2388	0.7520
	4	0.3930	0.2930	0.1106	0.2387	0.7600
	5	0.3609	0.2609	0.0964	0.2183	0.7300
	*Surge Phase (March 6 to July 1), T = 118*					
*γ*_*h*_	1	0.1000	0.0000	0.0000	0.0052	0.9720
	2	0.0995	-0.0005	0.0000	0.0056	0.9760
	3	0.0987	-0.0013	0.0000	0.0046	0.9760
	4	0.0987	-0.0013	0.0000	0.0044	0.9740
	5	0.0994	-0.0006	0.0000	0.0051	0.9920
*γ*_*v*_	1	0.1077	0.0077	0.0003	0.0192	0.9720
	2	0.1093	0.0093	0.0004	0.0190	0.9620
	3	0.1082	0.0082	0.0002	0.0163	0.9660
	4	0.1100	0.0100	0.0003	0.0164	0.9340
	5	0.1100	0.0100	0.0004	0.0201	0.9540

*Data Generation Mechanism

**Empirical Coverage Probability

We find that our method is able to accurately predict the non-ventilated census, ventilated census, and number of SARS-CoV-2 positive admissions for the upcoming 14 day period with a high degree of accuracy, even in the presence of model misspecification and/or a large number of undetected cases. The bias in the point estimators for these quantities is relatively small, and mean absolute out of sample prediction error is less than 20% in most cases. As expected, the performance of the model (measured by the mean percent absolute out of sample prediction error) improves as the pandemic progresses and more data is available to the model. Our method is able to accurately estimate the hospitalization recovery rate well, even when cases are underdetected and/or the model is misspecified. Estimation of the ventilation recovery rate is reliable only for the surge phase.

Assuming full case detection and correct model specification, the methodology accurately estimates the area-level case incidence. As expected, in the presence of underdetection, there is significant bias in area-level incidence estimates and the transmission rates (see Web Appendix B in S1 File for further discussion of these results). However, even when 100% of cases are detected, the empirical coverage intervals for the case recovery rate (*γ*_*i*_) and the area-level transmission rates (*β*_*c*_(*t*)*s*) are below their nominal levels, perhaps indicating a mild identifiabilty issue between these parameters. This is not surprising, given that an increase in the number of initially infected individuals can be offset by a decrease in the transmission rate or a increase in the recovery rate to produce the same number of incident infections (particularly during the early phases of a pandemic). Our primary goal is to predict the number of COVID-19 patients requiring inpatient care, not to perform inference with respect to model parameters. As the identifiability issue does not seem to impact the quantities of primary interest, it is of minimal concern for this application. We strongly caution the reader, however, against attempting to use this model to perform inference with respect to the recovery rate, transmission rate or the number of initially infected individuals.

A key strength of our method is that it accurately estimates the number of COVID-19 inpatients without requiring an accurate estimate of the true number of SARS-CoV-2 infections. Estimates of the proportion of SARS-CoV-2 infections which are asymptomatic vary, but several meta analyses place this number between 30% and 40% [32–34]. In the absence of extensive surveillance testing and/or contact tracing efforts, many of these asymptomatic cases go undetected. To avoid having to estimate undetected and asymptomatic cases, our method assumes transmission is driven by reported cases alone. The flexible spline-estimators of the transmission rate and the hospitalization rate allow the model to absorb the impact of the biased estimate of the number of infectious individuals which results from assuming simplified transmission dynamics. This bias is ‘upstream’ from the quantities of interest (hospital admissions, non-ventilated census, and ventilated census), and has a negligible effect on the these quantities, as evidenced by our simulation results. We see that our method accurately estimates the demand for inpatient care even when a large number of infections are unreported. We stress that this method is intended to predict hospitalized and ventilated cases only, and will not provide a reliable estimate of the true number of infections in presence of underreporting (see Web Appendix B for more details).

## Predictive assessment

In this section, we assess the predictive performance of the Bayesian SIHVR model for Prisma Health Upstate and Prisma Health Midlands. Since the two regional systems serve geographically distinct patient populations, we fit separate models to the data from each region. The model from the Upstate system included census and admissions data summed over all hospitals in the Prisma Upstate system, and county-level incidence and social distancing data from Greenville and Spartanburg counties. While Prisma Health Upstate serves patients across the Upstate of South Carolina, Greenville and Spartanburg counties are the most populous. The model for Prisma Midlands included census and admissions data summed over all hospitals in the Midlands system and county-level incidence and social distancing data from Kershaw, Lexington, Richland and Sumter counties. To ensure our predictive assessment was robust, we evaluated model performance during both South Carolina’s reopening phase (May, 2020), and first summer surge (June-July, 2020). Specifically, we fit the model using data from March 6th to May 1st, May 15th, June 1st, June 15th, and July 1st, for a total of 5 model fits per region. Each model was used to predict the daily reported case incidence, daily hospital admissions, daily non-ventilated census and daily ventilated census for the upcoming two week period. These predictions were compared to observed values to assess predictive performance. We are most concerned with the accuracy of short-term (14 day) predictions, as healthcare systems need these predictions to make immediate decisions regarding staffing, PPE allocation, and the feasibility of elective procedures. However, we also provide mid-range (28 day) projections in our results; such mid-range projections proved to be sufficiently accurate (i.e. within the 95% credible region) in most scenarios.

We evaluated three different methods for incorporating social distancing metrics into the transmission rate. Method 1 took ***z***_*ct*_ as the scalar *z*_*c*,*t*−14_, the USDS social distancing metric from day *t* − 14. Method 2 took ***z***_*ct*_ = (*z*_*c*,*t*−14_, *z*_*c*,*t*−13_, …, *z*_*c*,*t*−3_)′, thereby allowing *β*_*c*_(*t*) to be influenced by the social distancing metric from days *t* − 3 to *t* − 14. Method 3 allowed for a more complex relationship between past levels of the social distancing metric and *β*(*t*). Specifically, method 3 took $\beta_{c}\left(t\right)=\text{exp}\left\{x_{i_{c}}^{\prime }b_{i}+h_{c}\left(t\right)+\alpha_{c}\right\}$, where $h_{c}\left(t\right)=\sum_{i=t_{0}}^{t_{1}}f\left(z_{c,t-i},i\right)$, with *f*(*z*, *i*) being an unknown function that gives the influence of a social distancing level of *z* on day *t* − *i* on *β*(*t*). Note that *f*(*z*, *i*) is stationary with respect to *t*. The constants *t*_0_ and *t*_1_ are chosen reflect the range of past days whose social distancing metric affects *β*_*c*_(*t*), i.e., *β*_*c*_(*t*) is influenced by the amount of social distancing occurring between days *t* − *t*_1_ and *t* − *t*_0_, inclusive. The unknown function *f*(*z*, *i*) is approximated using the two dimensional tensor product of B-spline basis functions in the usual manner:

$$\begin{array}{c} f\left(z,i\right)\approx \sum_{k_{1}}^{K_{1}}\sum_{k_{2}}^{K_{2}}\phi_{k_{1}k_{2}}B_{1k_{1}}\left(z\right)B_{2k_{2}}\left(i\right) \end{array}$$

yielding

$$\begin{array}{c} h_{c}\left(t\right)=\sum_{i=t_{0}}^{t_{1}}f\left(z_{c,t-i},i\right)\approx \sum_{i=t_{0}}^{t_{1}}\sum_{k_{1}=1}^{K_{1}}\sum_{k_{2}=1}^{K_{2}}\phi_{k_{1}k_{2}}B_{1k_{1}}\left(z_{c,t-i}\right)B_{2k_{2}}\left(i\right) \end{array}$$

which implies

$$\begin{array}{c} z_{ct}={\left(\sum_{i=t_{0}}^{t_{1}}B_{11}\left(z_{c,t-i}\right)B_{11}\left(i\right),\sum_{i=t_{0}}^{t_{1}}B_{11}\left(z_{c,t-i}\right)B_{22}\left(i\right),\ldots ,\sum_{i=t_{0}}^{t_{1}}B_{1K_{1}}\left(z_{c,t-i}\right)B_{2K_{2}}\left(i\right)\right)}^{\prime }. \end{array}$$

For our analysis, we took *t*_0_ = 3, *t*_1_ = 14, *K*_1_ = *K*_2_ = 3, and allowed the $B_{1k_{1}}\left(\cdot \right)s$ and $B_{1k_{2}}\left(\cdots \right)$ to be cubic B-spline basis functions with 3 equally spaced knots. Method 1 allows one to forecast up to two weeks into the future without predicting future levels of social distancing, an advantage over the more complicated methods. Creating such forecasts with method 2 or 3 requires first predicting future levels of social distancing; these future values were predicted via last value carried forward.

Table 3 summarizes the 14-day predictive performance of our model with respect to non-ventilated census, ventilated census, and daily SARS-CoV-2 positive admissions for each of the 3 methods for incorporating social distancing. The table provides the mean absolute prediction error for each of these quantities (defined as the absolute difference between the observed quantity and its predicted median, averaged over all 14 days for which predictions were computed). We also provide the percent of predicted days for which the 95% highest posterior density prediction interval for each quantity captures the observed value. The average number of days for which the observed non-ventilated census fell within the 95% prediction interval were 99.29%, 92.29%, and 100% for methods 1, 2, and 3 respectively. The average number of days for which the observed ventilated census fell within the 95% prediction interval were 84.29.%, 87.14%, and 86.64% for methods 1, 2 and 3, with the 95% prediction intervals for the number of admissions capturing the observed values 97.86%, 95.71%, and 95.00% on days on average for the three methods. While no single method consistently had the smallest prediction error across quantities and models, Method 1 had the lowest total prediction error (summed over all quantities and models).

**Table 3:** The table provides the mean absolute prediction error and percent of observations falling with the 95% prediction interval (CP) for the non-ventilated census (NC), ventilated census (VC), and admissions (AD) for each of the 15 models fit to the data from the Upstate system (top) and Midlands system (bottom). Data from March 6th, 2020 to the day given in the Date column was used to fit each model, and the next 14 days of data was used to assess prediction.

Date	Method	NC Pred. Er	NC CP	VC Pred. Er	VC CP	AD Pred. Er.	AD CP
	*Upstate System*						
May 1	1	8.29	1.00	1.07	1.00	4.93	1.00
	2	3.14	1.00	0.64	1.00	2.93	1.00
	3	15.00	1.00	1.36	1.00	8.00	0.86
May 15	1	14.50	0.93	3.64	1.00	4.07	1.00
	2	10.14	1.00	3.43	1.00	2.71	1.00
	3	12.79	1.00	3.64	1.00	4.00	1.00
June 1	1	4.86	1.00	7.79	0.29	3.07	0.93
	2	8.36	1.00	8.07	0.29	3.71	0.86
	3	4.36	1.00	7.79	0.29	3.57	0.93
June 15	1	5.49	1.00	3.43	1.00	8.57	1.00
	2	12.00	1.00	4.00	1.00	4.50	1.00
	3	9.71	1.00	3.57	1.00	10.86	1.00
July 1	1	7.93	1.00	7.64	0.86	5.14	1.00
	2	42.50	1.00	6.07	1.00	19.14	1.00
	3	18.64	1.00	4.43	1.00	12.14	1.00
	*Midlands System*						
May 1	1	5.71	1.00	11.71	0.76	3.36	1.00
	2	25.29	0.29	4.50	1.00	2.71	0.79
	3	15.00	1.00	8.57	1.00	1.86	1.00
May 15	1	18.14	1.00	17.36	0.50	6.50	0.93
	2	22.86	1.00	18.93	0.43	7.64	1.00
	3	24.43	1.00	19.29	0.50	7.93	0.86
June 1	1	8.79	1.00	1.71	1.00	2.71	0.93
	2	9.43	1.00	1.64	1.00	2.79	0.93
	3	11.93	1.00	1.79	1.00	3.29	0.86
June 15	1	4.21	1.00	3.14	1.00	2.64	1.00
	2	3.71	1.00	2.64	1.00	2.86	1.00
	3	5.07	1.00	3.21	1.00	2.57	1.00
July 1	1	7.41	1.00	5.42	1.00	2.78	1.00
	2	8.50	1.00	5.43	1.00	3.00	1.00
	3	8.14	1.00	5.21	1.00	2.79	1.00

**Fig 4:**
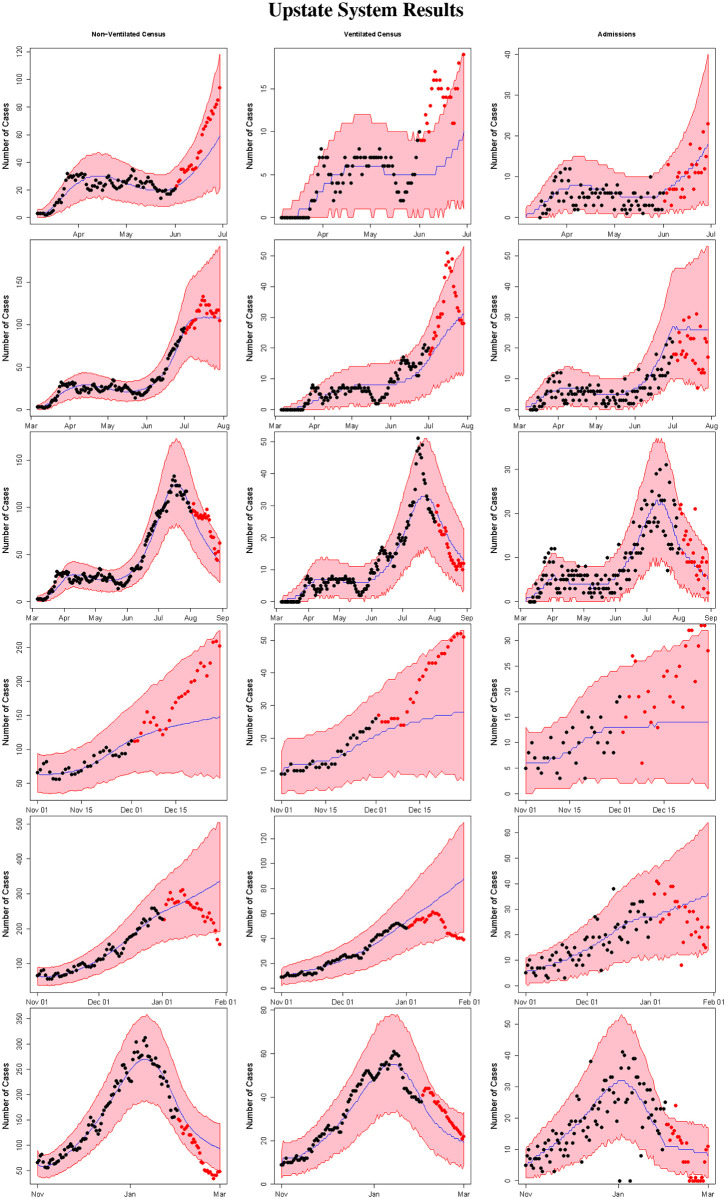
Upstate system results. The figure displays the results for the non-ventilated census (column 1), ventilated census (column 2), and SARS-CoV-2 positive admissions (column 3) for the Upstate system from the models fit using data from March 6th, 2020 to June 1st 2020 (row 1), July 1st 2020 (row 2), and August 1st 2020 (row 3), and November 1st, 2020 to December 1st, 2020 (row 4), January 1st 2021 (row 5), and February 1st 2021 (row 6). The red shaded regions denote 95% prediction intervals, the blue lines denote the median estimators, the black points denote the observed data used to fit the model, and the red points denote observed data from the 28 day forecast period (not used to fit the model).

**Fig 5:**
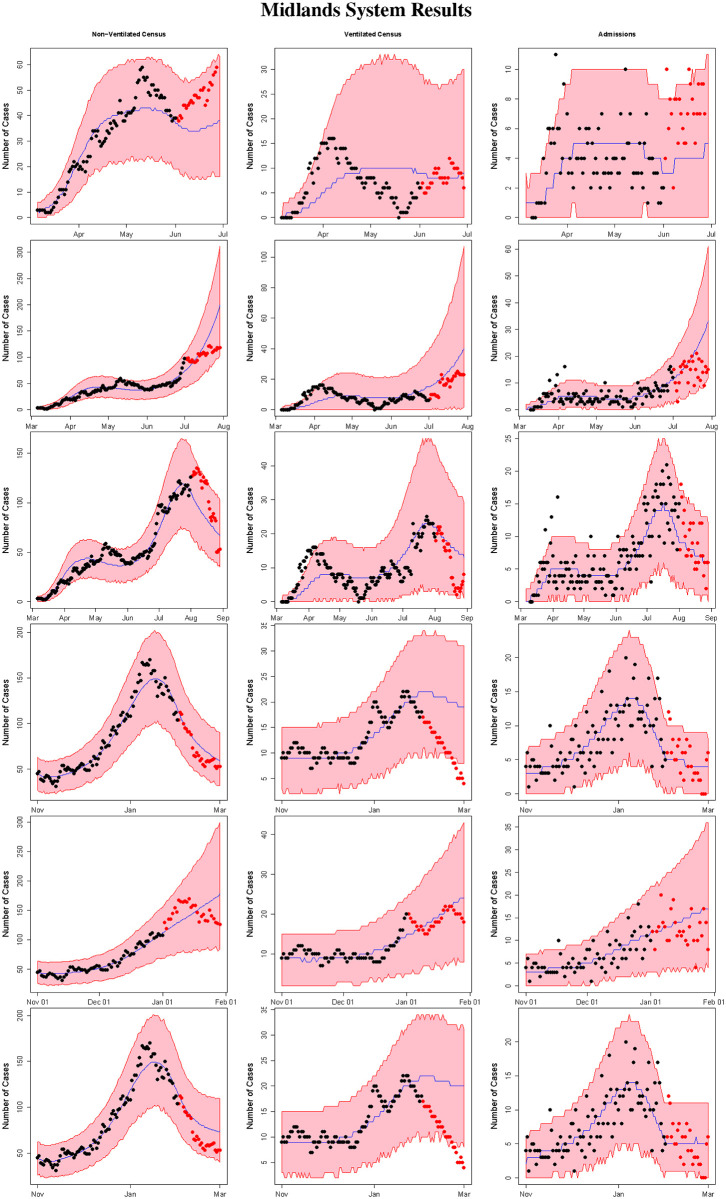
Midlands system results. The figure displays the results for the non-ventilated census (column 1), ventilated census (column 2), and SARS-CoV-2 positive admissions (column 3) for the Midlands system from the models fit using data from March 6th, 2020 to June 1st 2020 (row 1), July 1st 2020 (row 2), and August 1st 2020 (row 3), and November 1st, 2020 to December 1st, 2020 (row 4), January 1st 2021 (row 5), and February 1st 2021 (row 6). The red shaded regions denote 95% prediction intervals, the blue lines denote the median estimators, the black points denote the observed data used to fit the model, and the red points denote observed data from the 28 day forecast period (not used to fit the model).

Figs 4 and 5 summarize the results from applying method 1 during the summer and winter COVID-19 surges in the Upstate and Midlands, respectively. Specifically, the figures provide the median estimate, 95% prediction interval, and observed data for the non-ventilated census, ventilated census, and number of admissions from forecasts using data from March 6th 2020 to June 1st 2020, March 6th 2020 to July 1st 2020, March 6th 2020 to August 1st 2020, November 1st 2020 to December 1st 2020, November 1st 2020 to January 1st 2021 and November 1st 2020 to February 1st 2021. Comparing the model predictions to the observed data for the 28 day forecast periods reveals that the model generally provides accurate predictions, with performance improving as more and more data is provided to the model. Importantly, the prediction intervals accurately quantify the uncertainty in model predictions. South Carolina experienced a rapid surge in COVID-19 cases in June and early July 2020, as well as in December 2020 and early January 2021, with a corresponding in increase in COVID-19 hospitalizations [19]. Our model successfully predicted these surges in both the Upstate and the Midlands region. Web Figs 3 and 4 provide the estimates of the number of daily COVID-19 cases reported by each county in Upstate and Midlands System from each model fit (using method 1).

### Performance comparison

To further assess the performance of our methodology, we compared its performance to that of the Institute for Health Metrics and Evaluation (IHME) model. The IHME model provides national and state level estimates of daily COVID-19 deaths, COVID-19 cases, inpatient and ICU beds occupied by COVID-19 patients, and ventilators in use by COVID-19 patients. The initial IHME model was based based on a curve fitting technique rather than a standard epidemiological model and was widely criticized for its inaccuracy and non-traditional methods [8]. The IHME model was later revised to be based on a SEIR model [7]; we compare our model to this updated version. A major drawback of the IHME model, and most other well-known models, is that they are not locally customizable. The IHME model provides state-level projections, but no finer granularity. As a result, in order to compare our model to IHME, we fit our model using data aggregated over the entire state of South Carolina.

The number of reported COVID-19 cases in South Carolina was obtained from the New York Times COVID-19 GitHub repository [20]. The daily numbers of hospitalized COVID-19 patients and ventilated COVID-19 patients in were obtained from the COVID Tracking Project [35], and the daily numbers of newly admitted COVID-19 patients was obtained from the US Department of Health and Human Services [36]. The social distancing metric was Unacast’s ‘Change in Visits to Non-Essential Businesses’ metric for the entire state of South Carolina.

**Fig 6:**
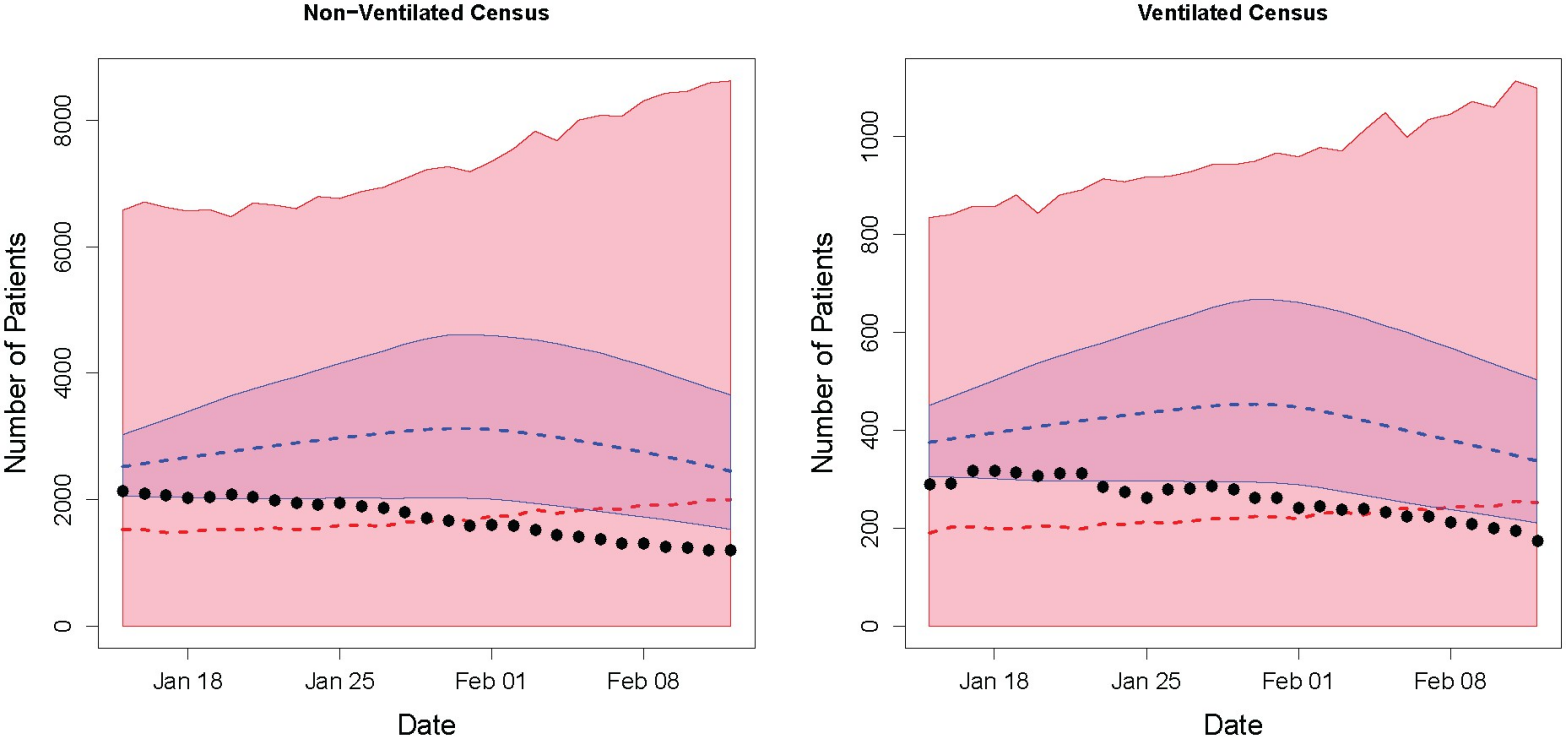
IHME comparison. The figure compares the performance of the Bayesian SIHVR model to the that of the IHME model. Depicted are the median (dashed red line) and 95% prediction interval (shaded red) from the Bayesian SIHVR model and the mean (dashed blue line) and 95% uncertainty interval (shaded blue) from the IHME model for the non-ventilated census (left) and ventilated census (right). Observed data points from the 28 day forecast period (not used to fit either model) are shown in black.

The functional forms of *β*_*c*_(⋅) and *ρ*_*h*_(⋅) and the prior distributions were identical to those described in the Methodology section, and method 1 was used to incorporate the social distancing data. However, here *C* = 1, and *U*_*ct*_, *W*_*t*_, *Y*_*t*_ and *Z*_*t*_ are the number of reported COVID-19 cases, COVID-19 hospital admissions, non-ventilated COVID-19 inpatients, and ventilated COVID-19 inpatients on day *t* for the entire state of South Carolina. We fit our model using data from November 1st, 2020 to January 15th, 2021, predicted out 28 days, and compared the results to the IHME’s reference model projections published on January 15th, 2021. Fig 6 displays the observed number of non-ventilated and ventilated COVID-19 patients in South Carolina for the 4 week forecast period starting on January 15th, along with the corresponding predictions from the Bayesian SIHVR model and the IHME model. While neither model is perfectly accurate, the Bayesian SIHVR has a clear advantage as the credible region for the IHME model does not capture the observed data for much of the forecast period. The IHME model has several strengths, including the fact that it produces estimates for every US state and many other countries using only data which is readily and widely available. This gives policy makers a way to easily compare predictions at different locations and allocate resources accordingly. However, the performance of our method shows that incorporating more (local) data sources can provide more accurate, locally tailored predictions for individual states or healthcare systems.

## Conclusion

The need to predict the burden of COVID-19 on local healthcare systems has persisted throughout the COVID-19 pandemic. The return to in-person instruction in schools and universities, the continued spread of more contagious SARS-CoV-2 variants such as the Delta variant, and uncertainty about the strength and duration of vaccine acquired immunity make the trajectory of the pandemic uncertain. The severity of the COVID-19 pandemic has displayed substantial heterogeneity among states, with Northeastern states such as New York and New Jersey being the hardest hit in the early stages of the pandemic, with Southern and Southwestern states such as Florida, Texas, Arizona and California seeing larger outbreaks during the summer 2020 and winter 2020–2021 surges. This heterogeneity makes it essential to produce reliable, locally customizable forecasts of COVID-19 burden so that resources can be transferred to where they are needed.

We have presented a method for forecasting the demand for inpatient COVID-19 care at the healthcare system level which is locally customizable. Our Bayesian SIHVR model incorporates local COVID-19 reported case incidence, local social distancing patterns, and healthcare system-specific COVID-19 data. Our model provides accurate short term (2–4 week) predictions and reliable regions of uncertainty. To facilitate the further use of our model by healthcare systems, code which fits the model has been developed in R and made available on GitHub (https://github.com/scwatson812/BayesianSIHVRModel).

As the scientific community continues to learn more about the epidemiology of COVID-19, the Bayeisan SIHVR model can be adapted to incorporate additional knowledge. For example, a ‘vaccinated’ state could be added to the model, with the rate of flow into this state estimated from data on the number of vaccines administered each day. The strength and duration of vaccine acquired immunity could be modeled by allowing individuals in the vaccinated state to become infected at a reduced rate, or to gradually return to the susceptible state over time as data emerges about the durability of vaccine-derived immunity. Reinfections could be modeled similarly by allowing recovered individuals to return to the susceptible state. As more knowledge is gained about the pervasiveness, contagiousness, and virulence of SARS-CoV-2 variants, additional infectious states could be added to represent different variants with different transmission and hospitalization rates. While it is not necessary to model every nuance in the epidemiological dynamics of COVID-19 in order to accurately predict the demand for COVID-19 inpatient care, the Bayesian SIHVR framework is flexible enough to allow researchers to include key information as it becomes available.

## Supporting information

S1 File Web appendix. The web appendix contains a description of the MCMC sampling algorithm (Web Appendix A), additional details regarding the simulation study (Web Appendix B), and additional details and figures regarding estimation of reported area-level COVID-19 case incidence with the Bayesian SIHVR model (Web Appendix C).
(PDF)

## References

[1] Centers for Disease Control and Prevention. Coronavirus (COVID-19) frequently asked questions. https://wwwcdcgov/coronavirus/2019-ncov/faqhtml. 2020;.

[2] Center for Systems Science and Engineering (CSSE) at Johns Hopkins University (JHU). COVID-19 Dashboard. https://coronavirusjhuedu/maphtml. 2020;. 10.1016/S1473-3099(22)00434-0 PMC9432867 36057267

[3] Oliver SE, Gargano JW, Marin M, Wallace M, Curran KG, Chamberland M, et al. The Advisory Committee on Immunization Practices’ Interim Recommendation for Use of Pfizer-BioNTech COVID-19 Vaccine—United States. MMWR Morbidity and mortality weekly report. 2020;69:1922–1924. 33332292 10.15585/mmwr.mm6950e2 PMC7745957

[4] Oliver SE, Gargano JW, Marin M, Wallace M, Curran KG, Chamberland M, et al. The Advisory Committee on Immunization Practices’ Interim Recommendation for Use of Moderna COVID-19 Vaccine—United States. MMWR Morbidity and mortality weekly report. 2020;69:1653–1656. 10.15585/mmwr.mm695152e1 PMC9191904 33382675

[5] Oliver SE, Gargano JW, Scobie H, Wallace M, Hadler SC, Leung J, et al. Advisory Committee on Immunization Practices’ Interim Recommendation for Use of Janssen COVID-19 Vaccine—United States. MMWR Morbidity and mortality weekly report. 2021;70:329–332. 33661860 10.15585/mmwr.mm7009e4 PMC7948932

[6] Attitudes Toward a Potential SARS-CoV-2 Vaccine. Annals of Internal Medicine. 2020;173(12):964–973. doi: 10.7326/M20-3569 32886525 PMC7505019

[7] Reiner RC, Barber RM, Collins JK, Zheng P, Adolph C, Albright J, et al. Modeling COVID-19 scenarios for the United States. Nature Medicine. 2020. doi: 10.1038/s41591-020-1132-9 PMC7806509 33097835

[8] Jewell NP, Lewnard JA, Jewell BL. Caution Warranted: Using the Institute for Health Metrics and Evaluation Model for Predicting the Course of the COVID-19 Pandemic. Annals of internal medicine. 2020;173(3):226–227. doi: 10.7326/M20-1565 32289150 PMC7197035

[9] Ray EL, Wattanachit N, Niemi J, Kanji AH, House K, Cramer EY, et al. Ensemble Forecasts of Coronavirus Disease 2019 (COVID-19) in the U.S. medRxiv. 2020. doi: 10.1101/2020.08.19.20177493

[10] Centers for Disease Control and Prevention. COVID-19 Forecasts: Deaths. https://wwwcdcgov/coronavirus/2019-ncov/covid-data/forecasting-ushtml. 2020. 34009769

[11] Weissman G, Crane-Droesch A, Chivers C, Luong T, Hanish A, Levy MZ, et al. Locally Informed Simulation to Predict Hospital Capacity Needs During the COVID-19 Pandemic. Annals of Internal Medicine. 2020. doi: 10.7326/M20-1260 PMC7153364 32259197

[12] Pei S, Shaman J. Initial Simulation of SARS-CoV2 Spread and Intervention Effects in the Continental US. medRxiv. 2020. doi: 10.1101/2020.03.21.20040303

[13] Dandekar R, Barbastathis G. Quantifying the effect of quarantine control in Covid-19 infectious spread using machine learning. medRxiv. 2020. doi: 10.1101/2020.04.03.20052084 PMC7671652 33225319

[14] Lemaitre JC, Grantz KH, Kaminsky J, Meredith HR, Truelove SA, Lauer SA, et al. A scenario modeling pipeline for COVID-19 emergency planning. medRxiv. 2020. doi: 10.1101/2020.06.11.20127894 PMC8024322 33824358

[15] De Brouwer E, Raimondi D, Moreau Y. Modeling the COVID-19 outbreaks and the effectiveness of the containment measures adopted across countries. medRxiv. 2020. doi: 10.1101/2020.04.02.20046375

[16] Mbuvha R, Marwala T. Bayesian inference of COVID-19 spreading rates in South Africa. PLOS ONE. 2020;15(8):1–16. doi: 10.1371/journal.pone.0237126 32756608 PMC7406053

[17] de Oliveira ACS, Morita LHM, da Silva EB, Zardo LAR, Fontes CJF, Granzotto DCT. Bayesian modeling of COVID-19 cases with a correction to account for under-reported cases. Infectious Disease Modelling. 2020;5:699–713. doi: 10.1016/j.idm.2020.09.005 32995681 PMC7513875

[18] Hidaka S, Torii T. Predicting Long-term Evolution of COVID-19 by On-going Data using Bayesian Susceptible-Infected-Removed Model. medRxiv. 2020. doi: 10.1101/2020.05.08.20094953

[19] South Carolina Department of Health and Environmental Control. South Carolina County-Level Data for COVID-19. https://scdhecgov/infectious-diseases/viruses/coronavirus-disease-2019-covid-19/south-carolina-county-level-data-covid-19;.

[20] Times TNY. NYT COVID-19 Data; 2021. https://github.com/nytimes/covid-19-data.

[21] Unacast. Unacast Social Distancing Dataset; 2020. https://www.unacast.com/data-for-good.

[22] Havers FP, Reed C, Lim T, Montgomery JM, Klena JD, Hall AJ, et al. Seroprevalence of Antibodies to SARS-CoV-2 in 10 Sites in the United States, March 23-May 12, 2020. JAMA Internal Medicine. 2020. doi: 10.1001/jamainternmed.2020.4130 32692365 PMC12507447

[23] Hao X, Cheng S, Wu D, Wu T, Lin X, Wang C. Reconstruction of the full transmission dynamics of COVID-19 in Wuhan. Nature. 2020;(584). doi: 10.1038/s41586-020-2554-8 32674112

[24] Dan JM, Mateus J, Kato Y, Hastie KM, Yu ED, Faliti CE, et al. Immunological memory to SARS-CoV-2 assessed for up to 8 months after infection. Science. 2021;371 (6529). doi: 10.1126/science.abf4063 33408181 PMC7919858

[25] Centers for Disease Control and Prevention. Reinfection with COVID-19. 2020;. 34009769

[26] De Boor C. A practical guide to splines. Springer-Verlag; 1978.

[27] Brauer F, van den Driessche P, Wu J. Mathematical Epidemiology. Springer-Verlag; 2008.

[28] Tolles J, Luong T. Modeling Epidemics With Compartmental Models. JAMA. 2020;323(24):2515–2516. doi: 10.1001/jama.2020.8420 32459319

[29] Guan Wj, Ni Zy, Hu Y, Liang Wh, Ou Cq, He Jx, et al. Clinical Characteristics of Coronavirus Disease 2019 in China. New England Journal of Medicine. 2020;382(18):1708–1720. doi: 10.1056/NEJMoa2002032 32109013 PMC7092819

[30] Kumar N, Shahul Hameed SK, Babu GR, Venkataswamy MM, Dinesh P, BG PK, et al. Descriptive epidemiology of SARS-CoV-2 infection in Karnataka state, South India: Transmission dynamics of symptomatic vs. asymptomatic infections. EClinicalMedicine. 2021;32:100717. doi: 10.1016/j.eclinm.2020.100717 33521608 PMC7831811

[31] Liu Y, Funk S, Flasche S. The contribution of pre-symptomatic infection to the transmission dynamics of COVID-2019. Wellcome Open Research. 2020;5(58). doi: 10.12688/wellcomeopenres.15788.1 PMC7324944 32685697

[32] Buitrago-Garcia D, Egli-Gany D, Counotte MJ, Hossmann S, Imeri H, Ipekci AM, et al. Occurrence and transmission potential of asymptomatic and presymptomatic SARS-CoV-2 infections: A living systematic review and meta-analysis. PLOS Medicine. 2020;17(9):1–25. doi: 10.1371/journal.pmed.1003346 32960881 PMC7508369

[33] Oran DP, Topol EJ. Prevalence of Asymptomatic SARS-CoV-2 Infection. Annals of Internal Medicine. 2020;173(5):362–367. doi: 10.7326/M20-3012 32491919 PMC7281624

[34] Oran DP, Topol EJ. The Proportion of SARS-CoV-2 Infections That Are Asymptomatic. Annals of Internal Medicine. 2021;174(5):655–662. 33481642 10.7326/M20-6976 PMC7839426

[35] Group TAM. The COVID Tracking Project. 2021.

[36] United States Department of Health and Human Services. COVID-19 Reported Patient Impact and Hospital Capacity by State Timeseries. 2021.

