# Supplementary material for: A Bayesian susceptible-infectious-hospitalized-ventilated-recovered model to predict demand for COVID-19 inpatient care in a large healthcare system
Source: PLoS One. 2022 Dec 15;17(12):e0260595. doi: 10.1371/journal.pone.0260595 (PMC9754233; doi:10.1371/journal.pone.0260595)
Supplement: S1 File — The web appendix contains a description of the MCMC sampling algorithm (Web Appendix A), additional details regarding the simulation study (Web Appendix B), and additional details and figures regarding estimation of reported area-level COVID-19 case incidence with the Bayesian SIHVR model (Web Appendix C). (PDF) [file pone.0260595.s001.pdf]

Web appendix for 'A Bayesian susceptible-infectious-hospitalized-ventilated-recovered model to predict demand for COVID-19 inpatient care in a large healthcare system'

Stella Coker Watson Self[1*], Rongjie Huang[1], Shrujan Amin[2], Joseph Ewing[2], Caroline Rudisill[1], Alexander C. McLain[1]

1 Department of Epidemiology and Biostatistics, Arnold School of Public Health, University of South Carolina, Columbia, South Carolina, United States of America
2 Care Coordination Institute, Prisma Health, Greenville, South Carolina, United States of America

*scwatson@mailbox.sc.edu (SCWS)

Web appendix A: Markov chain Monte Carlo algorithm for posterior sampling

Details of the Markov chain Monte Carlo algorithm used to sample from the posterior distribution of the Bayesian SIHVR model are provided in Algorithm 1. All parameters are sampled from their full conditional distributions with Metropolis Hastings steps. Evaluating the full conditional distribution each parameter requires solving the SIHVR system of differential equations using the most recently sampled values of all unknown parameters. The Euler method is used to provide a computationally efficient way to repeatedly solve the system of differential equations.

ALGORITHM 1: The Markov chain Monte Carlo algorithm used to fit the Bayesian SIHVR model. Here P_i, P_d, P_h, and P_v denote the dimensions of $\boldsymbol{b}_i$, $\boldsymbol{d}$, $\boldsymbol{b}_h$ and $\boldsymbol{b}_v$, respectively.

Initialize $\boldsymbol{\theta}^{(0)}$ for all unknown parameters $\boldsymbol{\theta}$;

for g $\leftarrow$ 1 **to** G
  for j $\leftarrow$ 1 **to** P$_i$
    Sample ${b_i}_j^{(g)}$;
  for j $\leftarrow$ 1 **to** P$_d$
    Sample $d_j^{(g)}$;
  Sample $\gamma_i^{(g)}$;
  Sample $\gamma_h^{(g)}$;
  Sample $\gamma_v^{(g)}$;
  for c $\leftarrow$ 1 **to** C
    Sample $\alpha_c^{(g)}$;
  for c $\leftarrow$ 1 **to** C
    Sample $I_c(0)^{(g)}$;
  for j $\leftarrow$ 1 **to** P$_h$
    Sample ${b_h}_j^{(g)}$;
  for j $\leftarrow$ 1 **to** P$_v$
    Sample ${b_v}_j^{(g)}$;
  Sample $\sigma_i^{(g)}$;
  Sample $\sigma_a^{(g)}$;
  Sample $\sigma_h^{(g)}$;
  Sample $\sigma_v^{(g)}$;

if $g \bmod 100 = 0$
then Tune the proposal distributions;

Web appendix B: Additional details regarding the simulation study

This section provides additional details regarding the simulation study described in Section 3 of the article. Under data generation mechanisms (DGMs) 3-5, data was generated from a susceptible-exposed-asymptomatic-presymptomatic-symptomatic-hospitalized-ventilated-recovered (SEAPSHVR) model. This model is specified by the following system of differential equations:

$$
\begin{aligned}
\frac{dS_c}{dt} &= -\beta_c(t)\{A_c(t) + P_c(t) + M_c(t)\}S_c(t)/N_c \\
\frac{dE_c}{dt} &= \beta_c(t)\{A_c(t) + P_c(t) + M_c(t)\}S_c(t)/N_c - \phi E_c \\
\frac{dA_c}{dt} &= \rho_a \phi E_c(t) - \gamma_a A_c(t) \\
\frac{dP_c}{dt} &= (1 - \rho_a)\phi E_c(t) - \tau P_c(t) \\
\frac{dM_c}{dt} &= \tau P_c(t) - \gamma_i M_c(t) - \rho_h(t) M_c(t) \\
\frac{dH_c}{dt} &= \rho_h(t) I_c(t) - \gamma_h H_c(t) - \rho_v(t) H_c(t) + \gamma_v V_c(t) \\
\frac{dV_c}{dt} &= \rho_v(t) H_c(t) - \gamma_v V_c(t) \\
\frac{dR_c}{dt} &= \gamma_a A_c(t) + \gamma_i M_c(t) + \gamma_h H_c(t)
\end{aligned}
$$

where $S_c(t)$, $E_c(t)$, $A_c(t)$, $P_c(t)$, $M_c(t)$, $H_c(t)$, $V_c(t)$ and $R_c(t)$ denote the number of susceptible, exposed, asymptomatic, presymptomatic, symptomatic, hospitalized, ventilated, and recovered individuals in area c at time t, respectively, ϕ is the rate at which exposed individuals become contagious (i.e. the reciprocal of the incubation period), ρ_a is the proportion of infections which are asymptomatic, γ_a is the recovery rate for asymptomatically infectious individuals, and τ is the rate at which presymptomatic individuals become symptomatic (i.e. the reciprocal of the presymptomatic infectious period).

Parameters for our simulation study were chosen so that the simulated data resembled the reported data for the Upstate system during various stages of the pandemic. For all 5 DGMs, we took $\boldsymbol{z}_{ct} = z_{ct-14}$, $\rho_v(t) = 0.05$, $\gamma_h = 1/10$, $\gamma_v = 1/10$, $\alpha_1 = 0$, $\alpha_2 = 0.1$ $N_1 = 498402$ and $N_2 = 302195$ (the populations of Greenville and Spartanburg counties). For each DGM, a piecewise cubic B-spline basis with 4 (3) equally spaced knots was used to estimate the transmission (hospitalization) rate. For DGMs 1 and 2, we assumed $\gamma_i = 1/14$ and that there was 1 initially infectious non-hospitalized individual in each county, 1.5 hospitalized individuals from each county (chosen to sum the total of 3 hospitalized individuals in the Upstate system on March 5th), 0 ventilated individuals in each county (chosen to sum the total of 0 ventilated individuals in the Upstate system on March 5th), and 0 recovered individuals in each county. The remaining individuals were assumed to be susceptible, giving the following initial conditions: $S_1(0) = 498399.5$, $S_2(0) = 302192.5$, $I_1(0) = I_2(0) = 1$, $H_1(0) = H_2(0) = 1.5$, $V_1(0) = V_2(0) = R_1(0) = R_2(0) = 0$. For DGMs 3-5, we assumed $S_1(0) = 498396.5$, $S_2(0) = 302189.5$, $E_1(0) = E_2(0) = A_1(0) = A_2(0) = P_1(0) = P_1(0) = M_1(0) = M_2(0) = 1$, $H_1(0) = H_2(0) = 1.5$, and $V_1(0) = V_2(0) = R_1(0) = R_2(0) = 0$. We also took $\phi = 1/3$, $\rho_a = 2/5$, $\gamma_i = 1/12$, $\gamma_a = 1/14$, and $\tau = 1/2$. In order to ensure that the simulated data displayed similar patterns to the real data from the Upstate system, it was necessary to specify different transmission and hospitalization rates for each DGM. Web Figures 1 and 2 show the values of $\beta_c(t)$, $c = 1, 2$, used for each simulation configuration and Web Figure 7 shows the values of $\rho_h(t)$ used for each simulation configuration.

Web appendix C: Additional details regarding estimation of reported COVID-19 cases

This section provides additional details regarding estimation of area-level reported case incidence using the Bayesian SIHVR model. While such estimation was not our primary goal, it may be of interest for healthcare systems to understand the local trajectory of the pandemic in their area. Web Table 1 provides the empirical bias, absolute prediction error, percent absolute prediction error, and 95% empirical coverage probability (ECP) for the reported case incidence from each area. The empirical bias is averaged over days $t = 1, 2, ...T$, and the other quantities are averaged over days $t = T + 1, T + 2, ..., T + 14$. As the posterior distribution of the reported incidence was right skewed, the posterior median was used as a point estimate. Empirical bias, absolute prediction error, and percent absolute prediction error were calculated with respect to the true case incidence, that is, with respect to the values of $\beta_c(t)I_c(t)S_c(t)/N_c$ in DGMs 1 and 2 and $\phi E_c(t)$ in DGMs 3, 4, and 5. However, 95% ECP was calculated using the *reported* case incidence, i.e., for $c = 1, 2$ and $t = T + 1, ..., T + 14$, U_{ct} was generated from a Poisson distribution with mean $\mu_{i_c,t}$, where $\mu_{i_c,t}$ is as specified in Section 3. ECPs were then calculated by assessing the how often the generated U_{ct} value fell within the corresponding 95% prediction interval. Web Table 2 provides the posterior mean estimate, empirical bias, MSE, standard deviation, and 95% ECP for the recovery rate γ_i. Web Figures 1 and 2 provide the posterior point estimate, true parameter value, and 95% prediction interval (averaged over all 500 datasets) for the reported case incidence and transmission rate for each area in the simulation for T = 57 and T = 118, respectively. The point estimators for the case incidence and transmission rate are the posterior median and mean, respectively.

Our simulation study found that the Bayesian SIHVR model was able to accurately estimate the daily number of new reported COVID-19 cases provided the model is correctly specified and there is no underreporting. In the presence of underdetection, the number of confirmed cases is a severely biased estimator of the true case numbers, and appears to underestimate them by a factor roughly comparable to the degree of underreporting present, as one would expect.

Web Figures 3 and 4 display the county-level reported case incidence results from applying the Bayesian SIHVR model (method 1) to data from the Upstate and Midlands Systems, respectively. The figures provide the posterior median (blue), reported case incidence used to fit the model (black), reported case incidence withheld to assess predictive performance (red), and 95% prediction intervals (shaded red). It is notable that the Bayesian SIHVR model accurately predicted both the summer and the winter surge for most counties, though the estimates for the November 1st to December 1st model appear to be unduly influenced by drastic changes in the social distancing metric over the Thanksgiving holiday and resulting erratic behavior of the transmission rate.

A note about the effective reproduction number

In infectious disease epidemiology, the effective reproduction number is commonly interpreted as the average number of new infections generated by each infectious person. The quantity has received much attention during the COVID-19 pandemic, and is commonly used to quantify the contagiousness of SARS-CoV-2 in a particular population at a particular point in time [1–3]. Under the standard suseptible-infectious-recovered (SIR) or suseptible-exposed-infectious recovered (SEIR) model, the effective reproduction number at time t is typically calculated as the transmission rate times the proportion of individuals who are susceptible at time t divided by the recovery rate [4]. Recall that the transmission rate is average the number contacts sufficient to spread the disease which an infected person has per day, and that the recovery rate is the reciprocal of the infectious period. The effective reproduction number can thus be thought of as the number of daily contacts sufficient to spread the disease times the number of days an individual is contagious times the proportion of those contacts which are susceptible. Applying this definition to our SIHVR model gives

$$R_c(t) = \frac{\beta_c(t)S_c(t)}{\gamma_i N_c}.$$

as the effective reproduction number in area c at time t. However, we note that in the context of the SIHVR model, this quantity should be interpreted with caution. In the typical SIR or SEIR model, the effective

reproduction number is greater than 1 if and only if the rate of change in the number of infectious individuals is positive. This is not the case in the SHIVR model, due to the flow from the infectious state to the hospitalized state. Therefore in the SHIVR model, it is possible for $R_c(t)$ to be greater than 1, but $I_c(t)$ to be decreasing. Additionally, when not all SARS-CoV-2 infections are reported, our simulation study demonstrates a large bias in the incidence of infection, which will translate to an biased estimate of $S_c(t)$, which will affect the estimate of $R_c(t)$. Secondly, in the absence of reliable data on the initial number of infections and/or the rate at which individuals are recovering from disease, $R_c(t)$ may not be fully identifiable, as changes in $S_c(t)$ may be offset by changes γ_i. We feel that any discussion of infectious disease transmission dynamics would be incomplete without consideration of the effective reproduction number, and therefore we include the posterior median estimates of $R_c(t)$ from each county from the models fit to data from March 6th, 2020 to August 1st, 2020 and November 1st 2020 to February 1st, 2021 in Web Figure 8. However we stress that given the identifiability problems identified in the simulation study, the SIHVR model should not be used to estimate $R_c(t)$ in the absence of additional data regarding the number of initially infected individuals and/or the recovery rate of individuals. Furthermore, in the SHIVR model, an $R_c(t)$ value less than 1 is not equivalent to a decreasing rate of infection, limiting its usefulness. If estimation of the effective reproduction number is of primary importance, a number of methods exists specifically for that purpose, including You et al. (2020) [5], Na et al. (2020) [6], and Medina-Ortiz et al. (2020) [7].

Web Table 1. Summary of Simulation Study Results: The table provides the empirical bias (averaged over days $1, 2, ..., T$ and the 500 datasets), empirical mean absolute prediction error (averaged over days $T + 1, T + 2, ..., T + 14$ and the 500 datasets), empirical mean percent absolute prediction error (averaged over averaged over days $T + 1, T + 2, ..., T + 14$ and the 500 datasets), and empirical coverage probability for 95% forecast prediction intervals (averaged averaged over days $T + 1, T + 2, ..., T + 14$ and the 500 datasets) for the reported case area-level case incidence.

**Quantity**	**DGM***	**Bias**	**Abs. Pred. Er.**	**% Abs. Pred. Er.**	**95% ECP****
		Early Phase (March 6 to May 1), T = 57			
	1	0.0027	9.7687	8.57	0.9751
	2	-66.4626	193.4928	89.63	0.9644
Area 1	3	-50.3651	406.1915	93.79	0.9516
	4	-6.1031	44.9578	47.50	0.9307
	5	-3.7177	24.9193	36.26	0.9397
	1	-0.0419	14.5346	8.34	0.9779
	2	-98.5124	295.8364	89.42	0.9636
Area 2	3	-42.4780	304.2462	93.47	0.9659
	4	-5.1659	32.9208	45.78	0.9491
	5	-3.1440	18.0265	34.62	0.9490
		Later Phase (March 6 to July 1), T = 118			
	1	0.0159	2.1869	4.28	0.9667
	2	-903.9671	722.7815	90.52	0.9371
Area 1	3	-1221.0096	1134.4837	94.01	0.8994
	4	-37.1752	30.5145	36.86	0.9093
	5	-50.5633	39.4418	0.2578	0.8933
	1	0.0363	3.9706	4.10	0.9733
	2	-987.0781	398.2774	87.21	0.7871
Area 2	3	-1189.1221	597.0883	91.55	0.7840
	4	-51.6570	47.8445	36.33	0.9167
	5	-67.7224	55.9333	0.2529	0.9077

*Data Generation Mechanism

**Empirical Coverage Probability

References

1. Arroyo-Marioli F, Bullano F, Kucinskas S, Rondón-Moreno C. Tracking R of COVID-19: A new real-time estimation using the Kalman filter. PLOS ONE. 2021;16(1):1–16. doi:10.1371/journal.pone.0244474.

2. Linka K, Peirlinck M, E EK. The reproduction number of COVID-19 and its correlation with public health interventions. Computational Mechanics. 2022;66:1035–1050. doi:https://doi.org/10.1007/s00466-020-01880-8.

3. Wilasang C, Sararat C, Jitsuk NC, Yolai N, Thammawijaya P, Auewarakul P, et al. Reduction in effective reproduction number of COVID-19 is higher in countries employing active case detection with prompt isolation. Journal of Travel Medicine. 2020;27(5). doi:10.1093/jtm/taaa095.

4. van den Driessche P. Reproduction numbers of infectious disease models. Infectious Disease Modelling. 2017;2:288–303. doi:doi:10.1016/j.idm.2017.06.002.

5. You C, Deng Y, Hu W, Sun J, Lin Q, Zhou F, et al. Estimation of the time-varying reproduction number of COVID-19 outbreak in China. International Journal of Hygiene and Environmental Health. 2020;228:113555. doi:https://doi.org/10.1016/j.ijheh.2020.113555.

6. Na J, Tibebu H, De Silva V, Kondoz A, Caine M. Probabilistic approximation of effective reproduction number of COVID-19 using daily death statistics. Chaos, Solitons Fractals. 2020;140:110181. doi:https://doi.org/10.1016/j.chaos.2020.110181.

7. Medina-Ortiz D, Contreras S, Barrera-Saavedra Y, Cabas-Mora G, Olivera-Nappa [U+FFFD] Country-Wise Forecast Model for the Effective Reproduction Number Rt of Coronavirus Disease. Frontiers in Physics. 2020;8:304. doi:10.3389/fphy.2020.00304.

Web Table 2. Summary of Simulation Study Results: The table provides the empirical bias (averaged over days $1, 2, ..., T$ and the 500 datasets), empirical mean absolute prediction error (averaged over days $T+1, T+2, ..., T+14$ and the 500 datasets), empirical mean percent absolute prediction error (averaged over averaged over days $T+1, T+2, ..., T+14$ and the 500 datasets), and empirical coverage probability for 95% forecast prediction intervals (averaged averaged over days $T+1, T+2, ..., T+14$ and the 500 datasets) for the reported case area-level case incidence.

Parameter	DGM*	Estimate	Bias	MSE	SD	95% ECP**
		Early Phase (March 6 to May 1), T = 57				
	1	0.0645	-0.0070	0.0006	0.0094	0.4640
	2	0.0540	-0.0174	0.0014	0.0135	0.4140
γ_i	3	0.0292	-0.0541	0.0036	0.0133	0.1800
	4	0.0486	-0.0347	0.0023	0.0143	0.3240
	5	0.0562	-0.0272	0.0019	0.0143	0.3800
		Later Phase (March 6 to July 1), T = 118				
	1	0.0710	-0.0004	0.0000	0.0022	0.5360
	2	0.0743	0.0029	0.0000	0.0023	0.4300
γ_i	3	0.0809	-0.0024	0.0001	0.0026	0.4200
	4	0.0807	-0.0027	0.0000	0.0025	0.4620
	5	0.0820	-0.0013	0.0000	0.0021	0.4840

*Data Generation Mechanism
**Empirical Coverage Probability

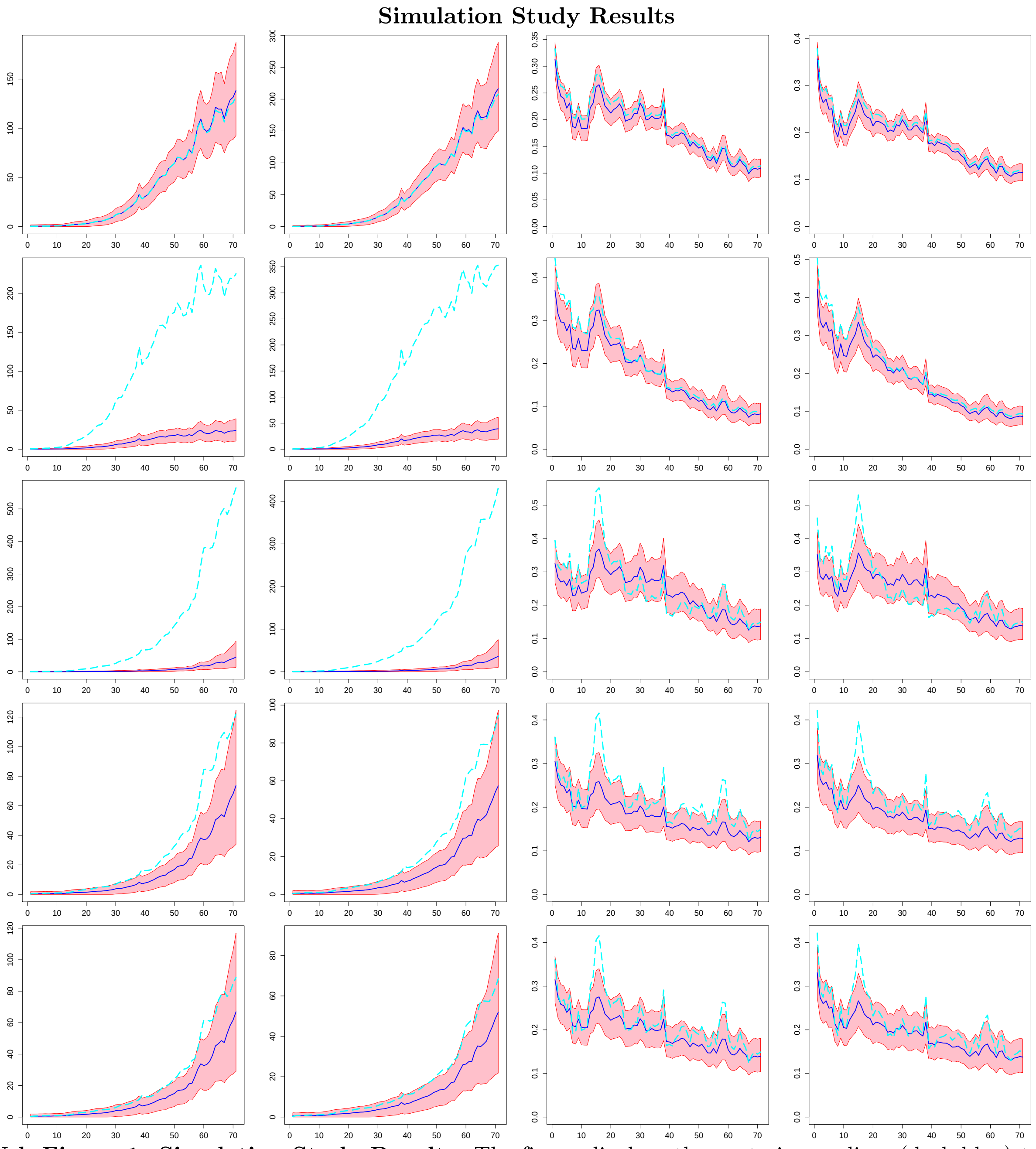

Web Figure 1. Simulation Study Results: The figure displays the posterior median, (dark blue) true value used for data generation, (light blue) and 95% credible interval (red) for the reported incidence in simulated geographic areas 1 and 2 (columns 1 and 2, respectively) and the posterior mean, (dark blue) true value used for data generation, (light blue) and 95% credible interval (red) for transmission rate in areas 1 and 2 (columns 3 and 4, respectively). From top to bottom, rows correspond to data generation mechanisms 1-5 with $T = 57$. In the presence of underdetection, the light blue line on the county incidence plots is the true number of cases, not the reported number.

Simulation Study Results

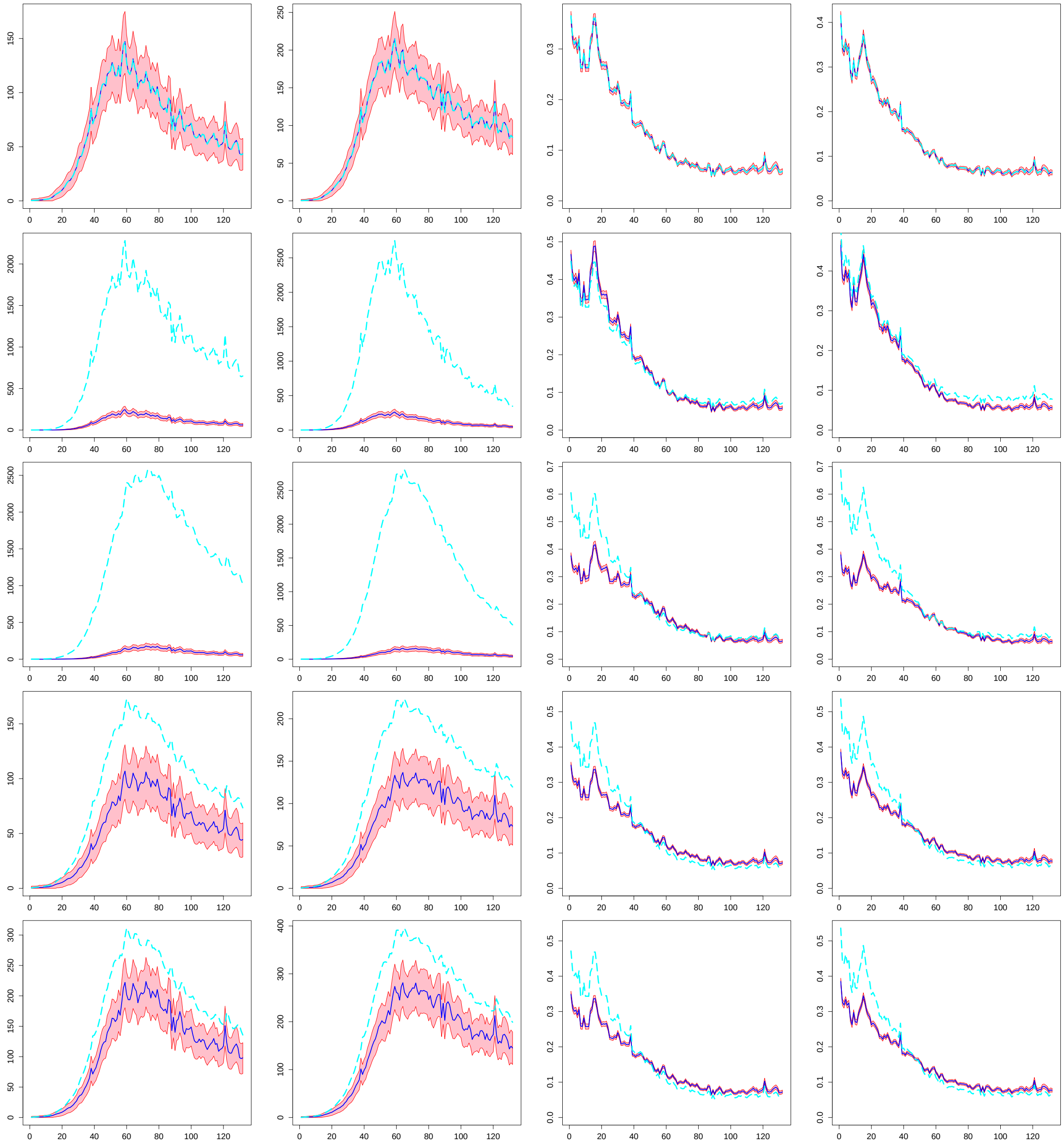

Web Figure 2. Simulation Study Results: The figure displays the posterior median, (dark blue) true value used for data generation, (light blue) and 95% credible interval (red) for the reported incidence in simulated geographic areas 1 and 2 (columns 1 and 2, respectively) and the posterior mean, (dark blue) true value used for data generation, (light blue) and 95% credible interval (red) for transmission rate in areas 1 and 2 (columns 3 and 4, respectively). From top to bottom, rows correspond to data generation mechanisms 1-5 with $T = 118$. In the presence of underdetection, the light blue line on the county incidence plots is the true number of cases, not the reported number.

Upstate System County Results

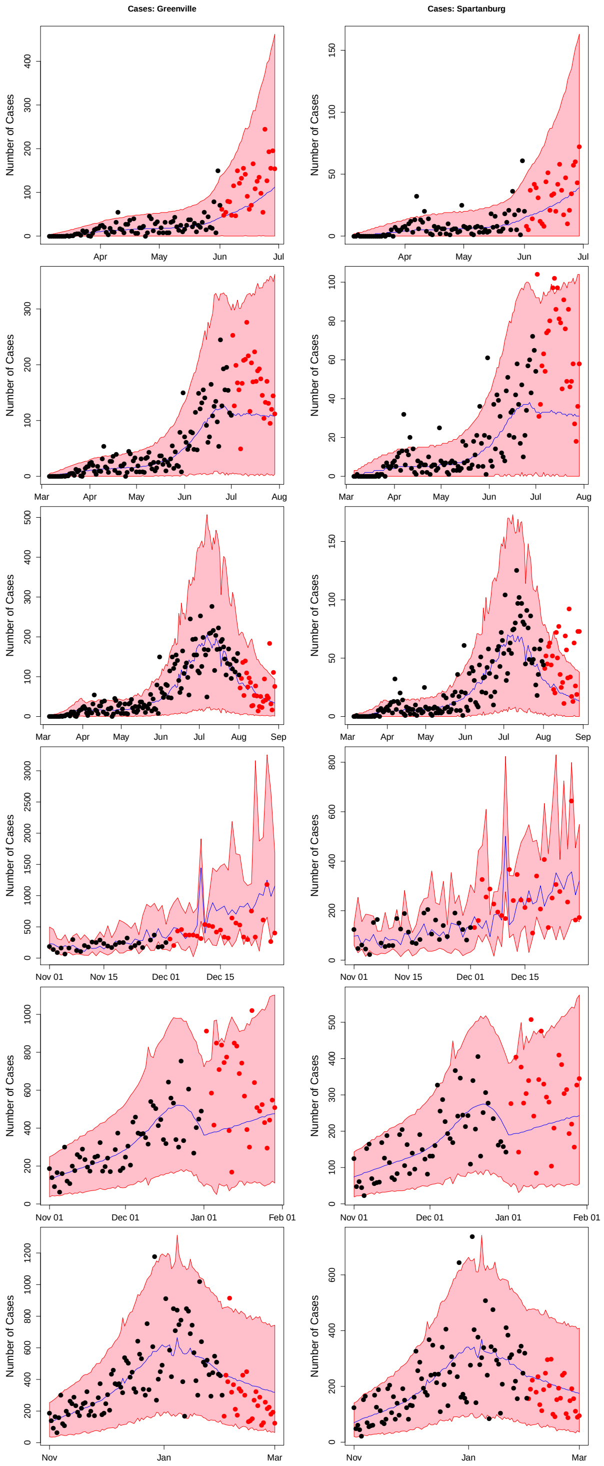

Web Figure 3. Upstate Case Results: The figure displays the model predicted number of reported COVID-19 cases for the counties in the Prisma Health Upstate System from the models fit using data from March 6th 2020 to June 1st 2020 (row 1), July 1st 2020 (row 2), August 1st 2020 (row 3), and November 1st 2020 to December 1st 2020 (row 4), January 1st 2021 (row 5) and February 1st 2021 (row 6). The red shaded regions denote 95% prediction intervals, the blue lines denote the median estimators, the black points denote the observed data used to fit the model, and the red points denote observed data from the 28 day forecast period (not used to fit the model)..

Midlands System County Results

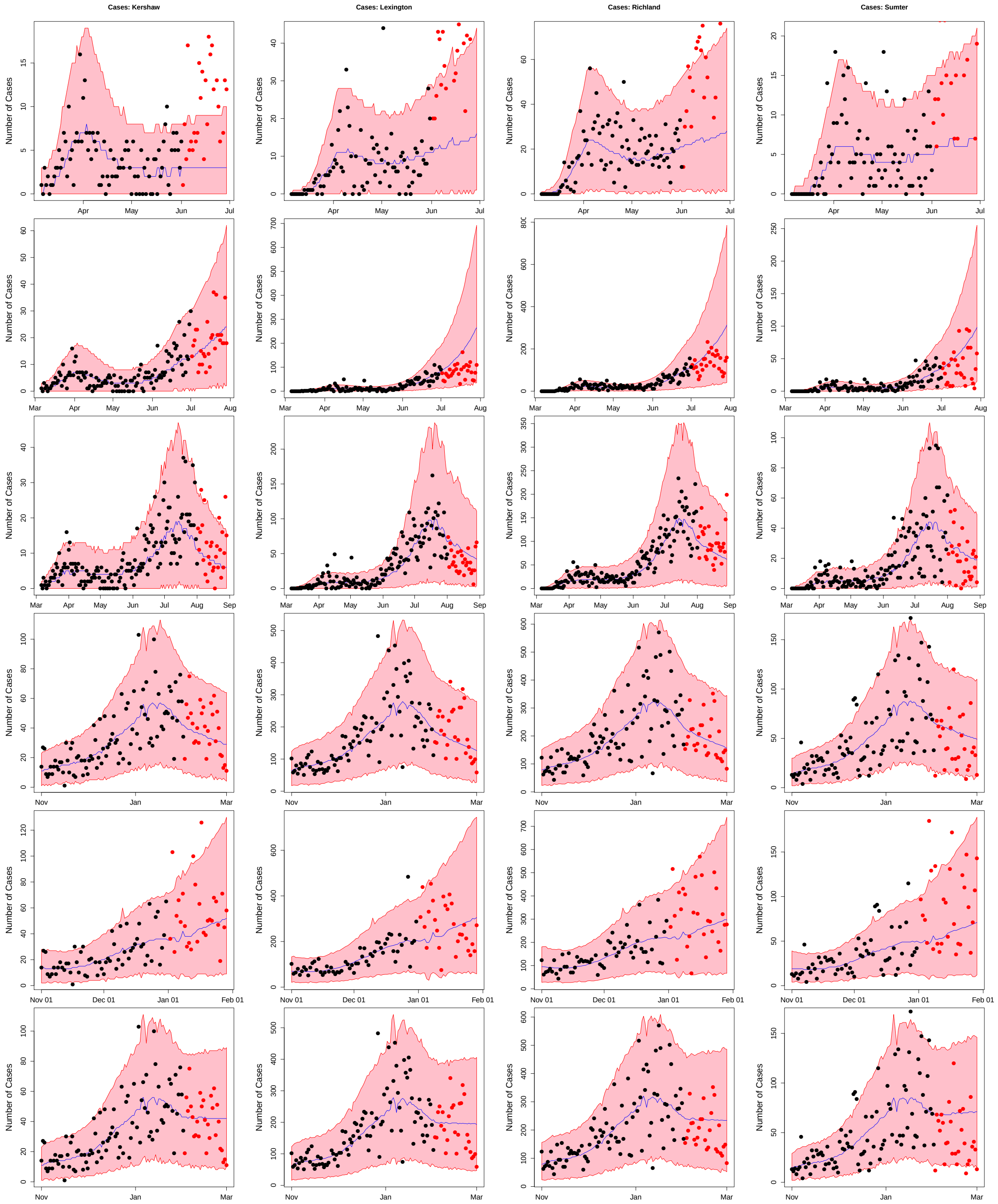

Web Figure 4. Midlands Case Results: The figure displays the model predicted number of reported COVID-19 cases for the counties in the Prisma Health Midlands System from the models fit using data from March 6th 2020 to June 1st 2020 (row 1), July 1st 2020 (row 2), August 1st 2020 (row 3), and November 1st 2020 to December 1st 2020 (row 4), January 1st 2021 (row 5) and February 1st 2021 (row 6). The red shaded regions denote 95% prediction intervals, the blue lines denote the median estimators, the black points denote the observed data used to fit the model, and the red points denote observed data from the 28 day forecast period (not used to fit the model).

Upstate System Transmission Rate Results

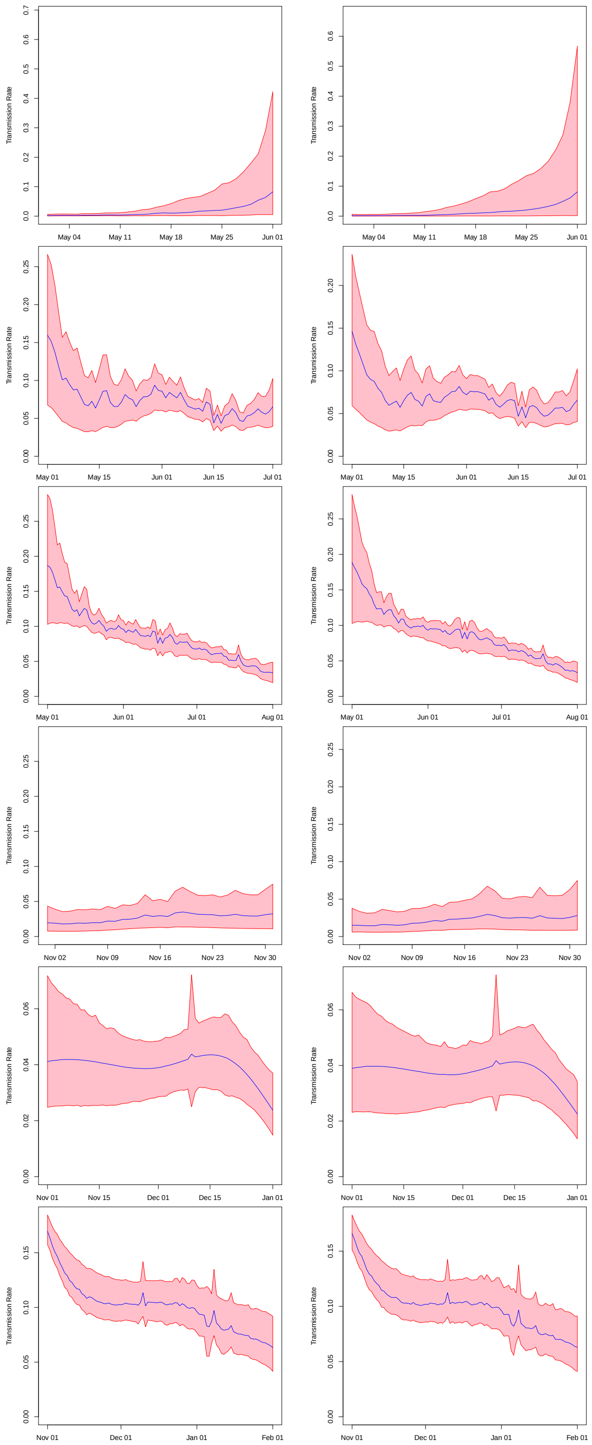

Web Figure 5. Upstate Transmission Results: The figure displays the model estimated transmission rate $\beta_c(t)$ for the counties in the Prisma Health Upstate System from the models fit using data from March 6th 2020 to June 1st 2020 (row 1), July 1st 2020 (row 2), August 1st 2020 (row 3), and November 1st 2020 to December 1st 2020 (row 4), January 1st 2021 (row 5) and February 1st 2021 (row 6). The red shaded regions denote 95% credible intervals, and the blue lines denote the posterior mean estimators.

Midlands System Transmission Rate Results

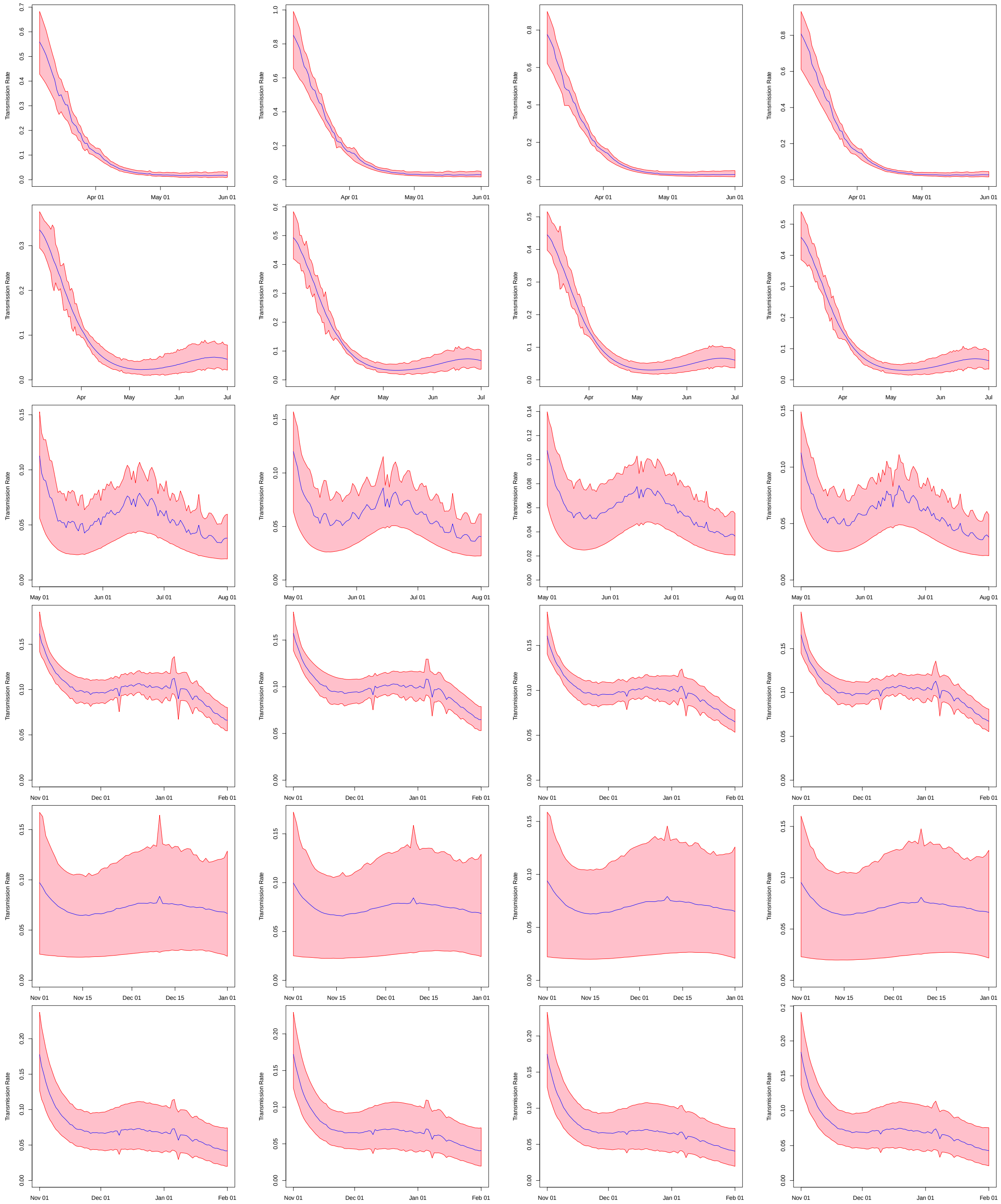

Web Figure 6. Midlands Transmission Results: The figure displays the model estimated transmission rate $\beta_c(t)$ for the counties in the Prisma Health Midlands System from the models fit using data from March 6th 2020 to June 1st 2020 (row 1), July 1st 2020 (row 2), August 1st 2020 (row 3), and November 1st 2020 to December 1st 2020 (row 4), January 1st 2021 (row 5) and February 1st 2021 (row 6). The red shaded regions denote 95% credible intervals, and the blue lines denote the posterior mean estimators.

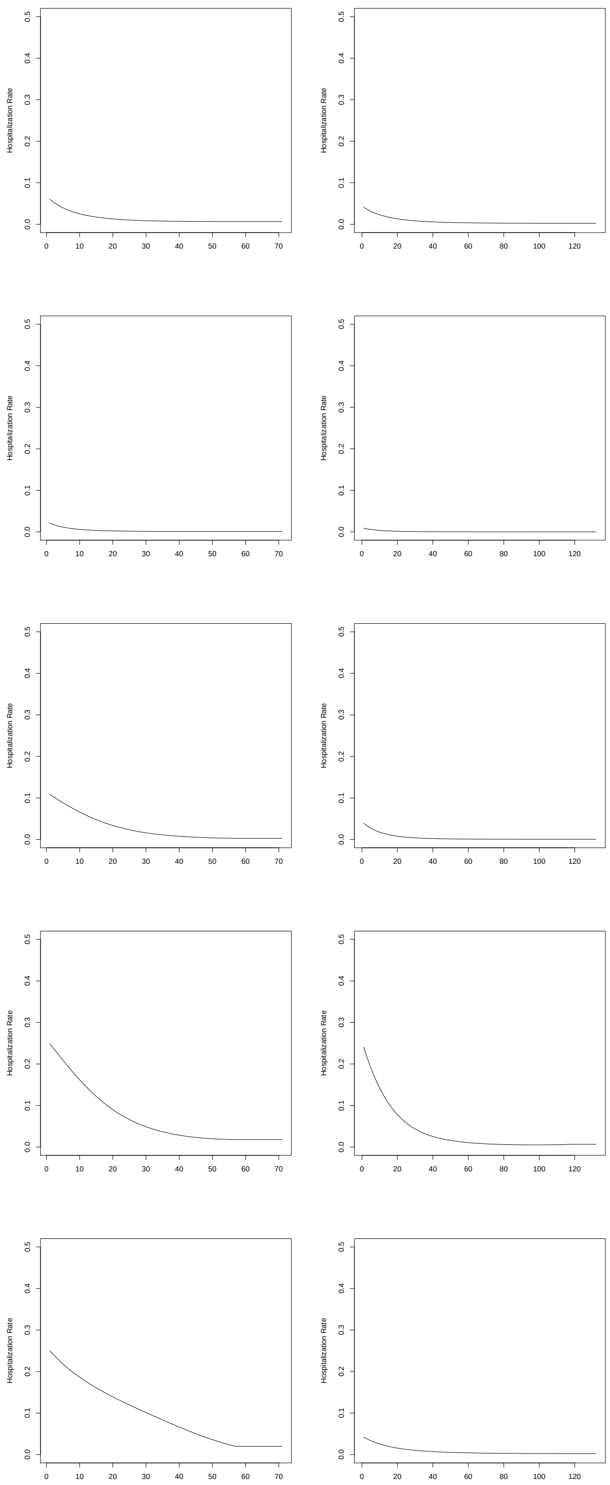

Web Figure 7. Hospitalization Rate: The figure displays the proportion of individuals in the infectious state entering the hospitalization state each day ($\rho_h(t)$) used for data generation in the simulation study. From top to bottom, the rows correspond to data generation mechanism 1-5 and the columns correspond to $T = 57$ (left) and $T = 118$ (right).

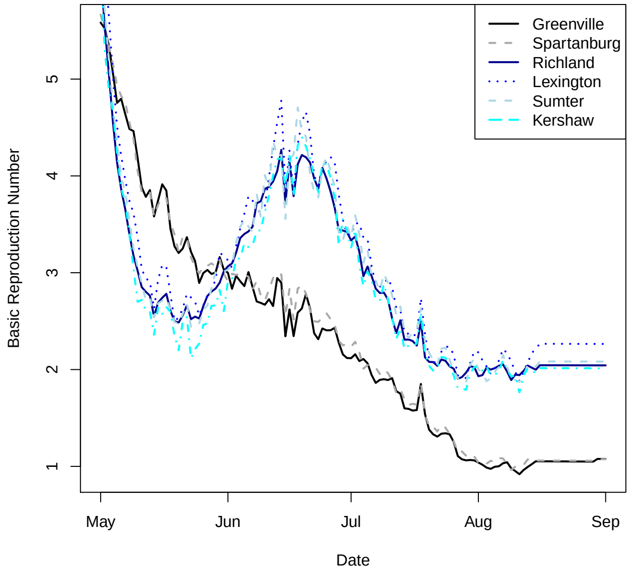

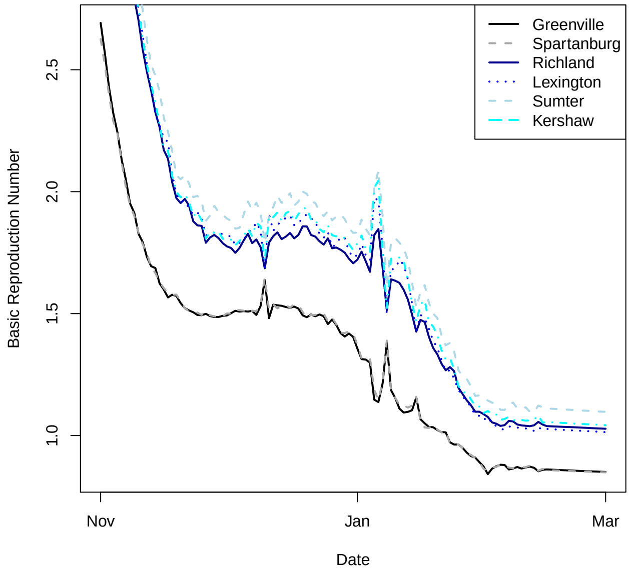

Web Figure 8. Effective Reproduction Number: The figure displays the posterior median estimate of the effective reproductive number $R_c(t)$ from Greenville, Spartanburg, Lexington, Richland, Sumpter, and Kershaw counties from the model fit to data from March 6th, 2020 to August 1st, 2020 (left) and November 1st, 2020 to February 1st, 2021 (right)
